# Observations on Spatial Specificity in the Modification of Porous Graphene Layers

**DOI:** 10.1002/cssc.202501031

**Published:** 2025-09-19

**Authors:** Abhijna Das, Marcus Waser, Kyoungjun Choi, Theodor Bühler, Christelle Jablonski, Aaron H. Oechsle, Junggou Kwon, Murray Height, Thomas A. Jung, Renzo A. Raso

**Affiliations:** ^1^ School of Life Sciences Institute of Chemistry and Bioanalytics University of Applied Sciences and Arts Northwestern Switzerland Hofackerstrasse 30 4132 Muttenz Switzerland; ^2^ Laboratory for X‐Ray Nanoscience and Technologies Center for Photon Science Paul Scherrer Institute Forschungsstrasse 111 5232 Villigen Switzerland; ^3^ HeiQ Materials AG Rütistrasse 12 8952 Schlieren Switzerland

**Keywords:** functionalization of graphene, graphene edge reactivity, porous graphene, self‐initiated photografting and photopolymerization, spatial specificity

## Abstract

Self‐initiated photografting and photopolymerization (SIPGP) is a simple one‐step polymerization process that can yield dense polymer layers on various surfaces, including pristine graphene. This process, however, has so far not been managed to be site selective. Herein, SIPGP is used to selectively functionalize the edges of pores in chemical vapor‐deposited porous graphene sheets. The pore edges formed during the graphene fabrication serve as directing reactive sites for the functionalization process. By polymerizing styrene monomers from the pore edges using a radical‐mediated process, polymer chains are preferentially grafted along and from the pore edges of the graphene. The spatial selectivity of the process is unambiguously demonstrated by the presence of a polymer rim around the pores in the atomic force microscopy data. The height of these polymer rims and the pore dimensions are measured, demonstrating the tunability of these characteristics by changing the reaction conditions (varying polymerization time from 0 to 24 h). The precise selectivity and controllability of the SIPGP process for the pore edges are potentially interesting for using porous graphene as functional membranes in different technological applications such as Per‐ and Polyfluoroalkyl substances (PFAs) ‐free waterproof membrane or state‐of‐the‐art membranes for water desalination.

## Graphical abstract

Exploiting the reactive defects at the pore edges, the possibility of regional‐specific functionalization is conclusively shown. This approach is simple, scalable, and facilitates the reinforcement of porous graphene. This targeted modification along the pore edges can lead to specific tailoring of the pore properties, offering the potential to fabricate the “ultimate graphene membrane.”

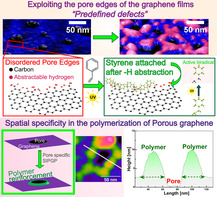

## 1 Introduction

Due to its excellent electrical,^[^ ^1^ ^]^ mechanical,^[^ ^2^ ^]^ and thermal properties,^[^ ^3^ ^]^ graphene is often dubbed the wonder material since it was first isolated using scotch tape.^[^ ^1^ ^]^ Starting from the methods established for fullerenes, graphene has been chemically modified extensively over the years.^[^ ^4^, ^5^ ^]^ The chemical stability of graphene, however, makes it very difficult to covalently functionalize the surface without generating defects in the graphene structure^[^ ^6^, ^7^, ^8^ ^]^ and disrupting the basal plane conjugation.^[^ ^9^ ^]^ For example, Sharma et al. employed diazonium reactant‐based electron transfer chemistry to attach functional groups at graphene edges;^[^ ^7^ ^]^ while impressive, they also reported an increased presence of *sp* ^3^‐hybridized carbons in the graphene samples with increasing grafting density.^[^ ^7^ ^]^ Another highly investigated functionalization approach involves the addition of dienophiles.^[^ ^10^ ^]^ However, those studies also reported a significant increase in the amount of *sp* ^3^ carbon species in the basal plane of the graphene surface.^[^ ^10^ ^]^ This has been deduced from the *I*_D_/*I*_G_ peak ratio from Raman spectra, in addition to their peak broadening after the reaction. Other covalent functionalization approaches include diazonium‐mediated aryl additions, Diels‐Alder‐mediated cycloaddition reactions, or in some cases, the introduction of single atoms via, e.g., plasma‐based treatments.^[^ ^4^, ^5^, ^10^ ^]^ While these functionalization processes are effective, they have several limitations, including poor site selectivity,^[^ ^4^, ^5^, ^10^ ^]^ harsh reaction conditions,^[^ ^4^, ^5^, ^10^ ^]^ and, perhaps more importantly, the disruption of the *sp* ^2^ conjugation in the basal plane^[^ ^4^, ^5^, ^10^ ^]^ that can worsen the properties of the graphene layer.^[^ ^11^ ^]^ Hence, Jordan and co‐workers first employed a self‐initiated photografting and photopolymerization (SIPGP) method to grow dense layers of polymers from pristine single‐layer graphene^[^ ^12^ ^]^ to circumvent these issues. SIPGP is a well‐known and widely used initiator‐free polymerization technique that utilizes the monomer as the photosensitizer.^[^ ^13^, ^14^, ^15^, ^16^ ^]^ Since then, many studies have reported the use of SIPGP to modify graphene or graphene oxide with target applications for electronics,^[^ ^17^, ^18^ ^]^ membranes,^[^ ^19^, ^20^ ^]^ and sensing.^[^ ^21^, ^22^, ^23^ ^]^

The pivotal advantage of the SIPGP process lies in the ability to selectively target and modify only the hydrogen‐passivated defect sites instead of the basal plane.^[^ ^24^, ^25^, ^26^, ^27^ ^]^ Previous studies on modified graphene have used methods like plasma^[^ ^28^ ^]^ or UV treatment of pristine single‐layer graphene with the presence of a photomask^[^ ^12^ ^]^ to specifically generate patterned defects on the graphene surface, serving as the starting point for SIPGP. These studies showed the possibility for polymer chains to be initiated at patterns of defects using the SIPGP method. The resolution of these patterns was limited to tens of micrometers.^[^ ^12^, ^18^, ^29^, ^30^, ^31^ ^]^ This resolution limit relates to the preprocessing steps, which often involve the use of a photomask (during UV light exposure)^[^ ^12^, ^18^ ^]^ or electron beam carbon deposition.^[^ ^12^, ^30^ ^]^ These UV‐mediated photolithography techniques have diffraction‐limited resolution over the modification areas.^[^ ^32^ ^]^ Improving the resolution of the patterned polymer brushes down to the nanometer scale requires introducing complex and costly methods such as e‐beam lithography or tip‐induced patterning, among others.^[^ ^31^ ^]^

Our present work addresses this issue by circumventing the preprocessing steps necessary for the selective grafting of polymers by introducing and utilizing predefined defects in the form of pores during the synthesis of the graphene itself. The location‐specific modification of graphene at pore sites may open a pathway to many membrane applications, specifically for chemical vapor‐deposited (CVD) porous graphene. Note that the utilized approach for the precise and efficient site‐specific grafting of polymer builds on earlier work exploiting the enhanced reactivity of graphene edges^[^ ^33^ ^]^ to attach nanoparticles selectively,^[^ ^34^ ^]^ functional molecules,^[^ ^7^ ^]^ and atomic layer deposited metal oxides.^[^ ^35^ ^]^

Widely cited as the ultimate membrane material,^[^ ^36^, ^37^ ^]^ porous graphene has gained significant interest in the last decade due to its potential in water desalination,^[^ ^37^ ^]^ gas separation,^[^ ^38^ ^]^ and other challenging membrane applications.^[^ ^39^, ^40^ ^]^ The mechanical stability of CVD porous graphene is still a matter of contention, partly due to the defect sites at the pore edges.^[^ ^41^, ^42^ ^]^ The pore edges are often dynamic as they are merely dangling bonds incompletely passivated by hydrogen atoms.^[^ ^43^, ^44^, ^45^, ^46^ ^]^ Previous studies have shown that these pore edges are prone to alteration or reconstitution over time.^[^ ^47^, ^48^, ^49^, ^50^ ^]^ Recent studies have shown that external forces can expand or shrink graphene pores.^[^ ^48^, ^49^ ^]^ Note that the passivation of the pore edges with Si atoms can make the pores resistant to such alterations.^[^ ^50^ ^]^ A further mechanism affecting pore sizes of CVD graphene is the interaction with water molecules, for example, during processing. It has been demonstrated that CVD graphene films can be ruptured due to sub‐critical crack growth and propagation due to stress corrosion in the presence of water.^[^ ^51^ ^]^ This is of particular concern as the processing steps of these films often include wet etching steps to remove the metal catalyst.^[^ ^41^, ^42^, ^52^, ^53^ ^]^ Different strategies to minimize cracking have been proposed, including stacking multiple graphene layers^[^ ^41^ ^]^ and interfacial polymerization after transfer.^[^ ^52^ ^]^ Robust passivation strategies of pore edges are needed to prevent their undesired reconstitution by extended exposure to the atmosphere.^[^ ^54^ ^]^

In brief, right after CVD synthesis, both pristine graphene and porous graphene remain reactive until the defective sites relax by different mechanisms, such as reorientation (*i.e.*, formation of new and more stable chemical bonds in the basal plane)^[^ ^44^, ^46^, ^54^ ^]^ or by chemical passivation, depending on the environmental conditions and time.^[^ ^44^, ^46^, ^54^ ^]^ The latter process is of particular importance as the reactive edges may be passivated with hydrogen or oxygen atoms after exposing to H_2_ gas, moisture, and aqueous etchants.^[^ ^51^ ^]^ Due to this intrinsic instability right upon synthesis, however, the graphene material is prone to relaxation‐mediated rupture, especially during the removal of the metal catalyst via etching processes.^[^ ^41^, ^42^, ^52^, ^53^ ^]^ Pore‐site selective functionalization stabilized mechanically and chemically the graphene layers.

We have used porous graphene produced by CVD in a bottom‐up approach^[^ ^55^ ^]^ that exploits a self‐assembly process^[^ ^56^, ^57^ ^]^ rather than traditional top‐down approaches such as focused ion beam, electron beam milling,^[^ ^36^, ^58^, ^59^ ^]^ chemical treatment,^[^ ^52^, ^60^ ^]^ and lithographic methods that utilize block copolymers.^[^ ^61^, ^62^ ^]^ Compared to the porous graphene prepared by top‐down techniques, the bottom‐up route can produce porous graphene with very few defects in the basal plane,^[^ ^55^ ^]^ forming reactive defect areas in a predefined way depending on the growth conditions. In this bottom‐up fabrication process, we modified the porous graphene with SIPGP, seeking to exploit the reactive sites created by CVD for further functionalization. Here, we have conclusively outlined a simple route to selectively modify the reactive pore edges, demonstrating the possibility of fabricating polymer‐reinforced porous graphene layers for future high‐performance membranes.

## 2 Results and Discussion

When synthesizing porous graphene in a reductive process, the pores are structural defects characterized by hydrogen‐terminated carbon atoms. The detailed process of the graphene fabrication method used in this study has been described earlier.^[^ ^55^ ^]^ The pores are formed by selectively inhibiting graphene growth via the presence of sacrificial tungsten particles on the copper‐based metal catalyst (see S1 and S7, Figure S9B,C, Supporting Information).^[^ ^55^ ^]^ The tungsten acts as a carbon sink, yielding tungsten carbide. In the surroundings of the tungsten particles, the graphene layer (basal plane) is free to grow. The catalyst size determines the lateral extension of the graphene basal plane and can reach several centimeters in size (much larger than graphene flakes); the graphene grains merge completely during synthesis (Figure S9B,C, Supporting Information).^[^ ^55^ ^]^ Before graphene synthesis, the tungsten particles are formed during the annealing process of a very thin tungsten film predeposited on the metal catalyst, which follows a spinodal dewetting mechanism.^[^ ^56^, ^63^ ^]^ Hence, the pore sizes and density can be controlled by varying the tungsten film thickness^[^ ^64^ ^]^ (within a range) as well as the annealing temperature^[^ ^65^ ^]^ and C‐source partial pressure (e.g., methane).^[^ ^55^ ^]^ Here, we have used a constant tungsten film thickness (4 nm) and identical annealing conditions to ensure similar porosities in the resulting graphene layers.

**Figure 1:**
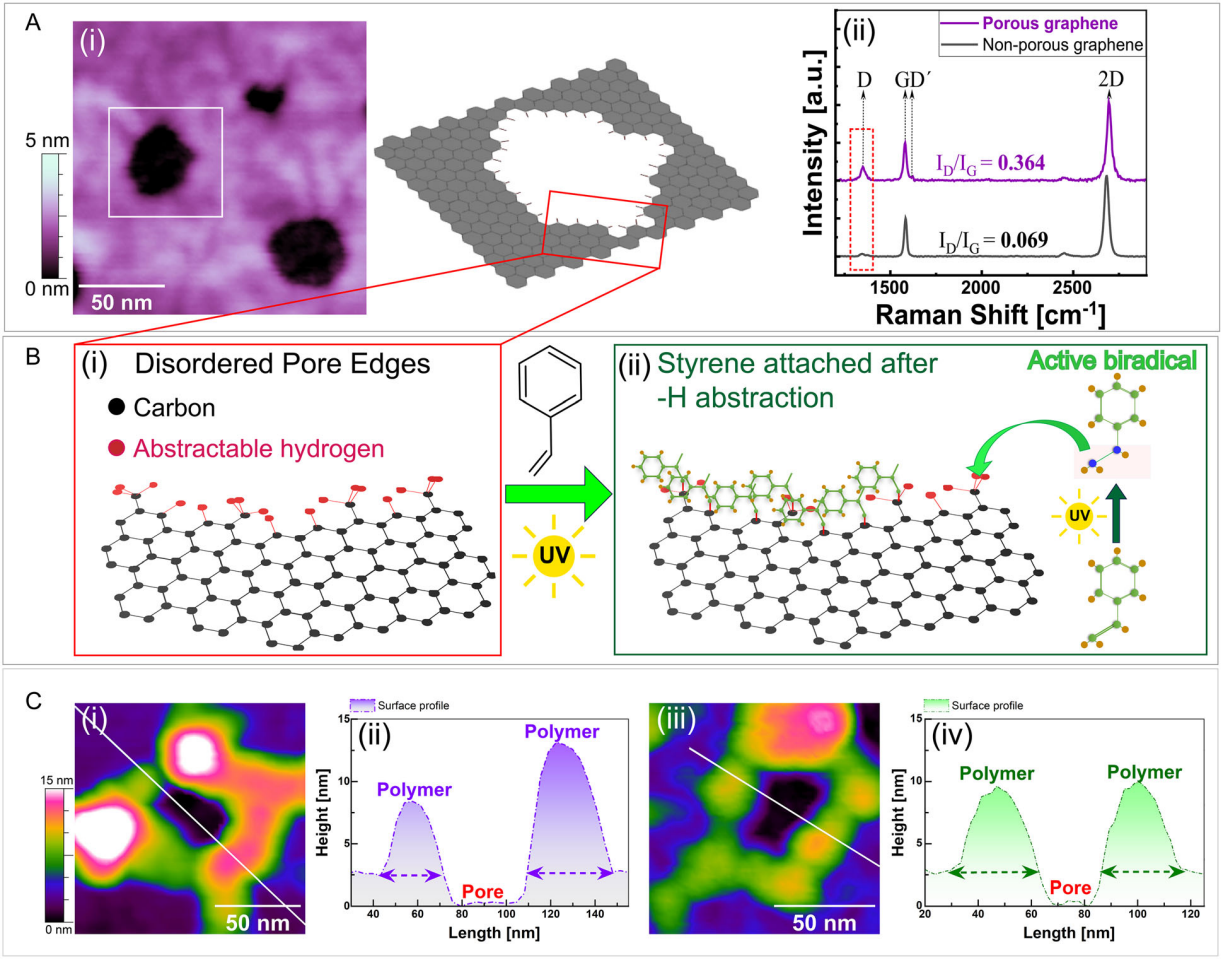
Site specificity of SIPGP on porous graphene. A) (i) Representative AFM image (scale bar = 50 nm, height range = 5 nm) of a porous graphene film transferred onto a silicon substrate. The schematic diagram shows the idealized (not in scale) representations of the edges of the graphene pores with both zigzag and armchair orientations. (ii) The Raman spectra of a single‐layer region of the porous graphene compared to a nonporous pristine graphene. The *I*_D_/*I*_G_ types are indicated in the plot. B) Schematic drawing depicting the SIPGP process on the porous graphene illustrating the mechanism of the grafting process at the defects and pore edges. (i) The schematic diagram of an edge (armchair and zig/zag configuration) passivated by hydrogen atoms. (ii) Styrene is attached at the edges after hydrogen abstraction, reacting with activated styrene biradical monomer^[^ ^12^, ^13^, ^30^, ^89^ ^]^ and UV light, leading to the polymerization of styrene at the pore edges. For a detailed mechanistic explanation of the styrene biradical‐mediated grafting, please see literature.^[^ ^13^, ^89^ ^]^ C) AFM images and surface profiles of pores after 4 h of SIPGP with styrene. All the samples were washed thoroughly with solvents before characterization. (i) and (iii) AFM images of the pore edges showing polymer domains grafted along the pore edges. (ii) and (iv) The surface profile of the pores along the lines drawn in (i) and (iii) depicts the presence of polymer domains with lateral resolution in the order of tens of nanometers.

During or right after the bottom‐up fabrication process, we expect the pores to have numerous dangling bonds at their edges, leading to predominantly C—H moieties formed by hydrogen passivation (**Figure** **1**Ai).^[^ ^43^, ^44^, ^45^, ^46^ ^]^ The Raman analysis (Figure 1Aii) of this type of porous graphene confirmed the emergence of both D and D´ peaks with *I*_D_/*I*_G_ and *I*_D_/*I*_D´_ ratios being 0.364 and 3.309, respectively. According to prior art, a D peak^[^ ^66^ ^]^ and the *I*_D_/*I*_D´_ ratio value of 3^[^ ^55^, ^67^ ^]^ are observed in the Raman spectra when disordered edges^[^ ^66^ ^]^ are present in graphene layers rather than vacancy‐type defects. Previous studies focusing on graphene edges have also reported that the dangling bonds at graphene edges often get passivated by hydrogen atoms at ambient temperature.^[^ ^43^, ^44^, ^45^, ^46^ ^]^ Therefore, in this scenario, the pore edges can be rationalized as nonuniform or disordered with abundant C—H groups attached to *sp* ^2^ and *sp* ^3^ hybridized carbon.

While the graphene basal plane can itself contain other defects such as grain boundaries, structural defects such as vacancies, *sp* ^3^ carbons contained in the *sp* ^2^ hybridized carbon matrix, and many others,^[^ ^43^, ^44^, ^45^, ^46^ ^]^ we assume that the predominant defects in these surfaces are pores that mainly contain abstractable hydrogen atoms, with few oxygen‐containing functionalities such as —OH, and —COOH introduced during different processing steps, including wet etching process with ammonium persulfate.^[^ ^33^, ^44^, ^68^ ^]^ SIPGP was used to functionalize the porous graphene surface (Figure 1B; see Supporting Information). As the work by Seifert et al. points out, the growth of polymer chains via the SIPGP process with styrene or styrene derivatives requires only the presence of abstractable hydrogen species, e.g., from C—H or CH_3_ moieties.^[^ ^12^, ^28^, ^30^ ^]^ Based on the enhanced intensity of the D peak in the Raman analysis of the films, the pore edges of the porous graphene films used in this study should present such sites.^[^ ^66^ ^]^ Previous studies describe the use of SIPGP to modify hydrogenated graphene^[^ ^28^ ^]^ and native *sp* ^3^‐hybridized carbon defects.^[^ ^12^, ^18^ ^]^ In SIPGP grafting, UV‐activated monomers (activated biradicals) abstract hydrogen atoms, followed by surface‐mediated polymerization (see Figure 1B).^[^ ^12^, ^18^, ^28^ ^]^ The polymerization would be confined to the defects in the graphene films as they expose suitable hydrogen atoms. Hence, instead of polymer carpets observed in other studies,^[^ ^12^, ^18^, ^28^ ^]^ we observe domains of collapsed polymer grafted along the pore edges due to constrained grafting areas (Figure 1C).^[^ ^69^, ^70^ ^]^ Figure 1C shows the atomic force microscopy (AFM) images of the pore morphology after 4 h of styrene via SIPGP. The AFM images revealed a thickening of the layer, especially along the pore edges, supporting the previously made assumption for spatially selective grafting.

**Figure 2:**
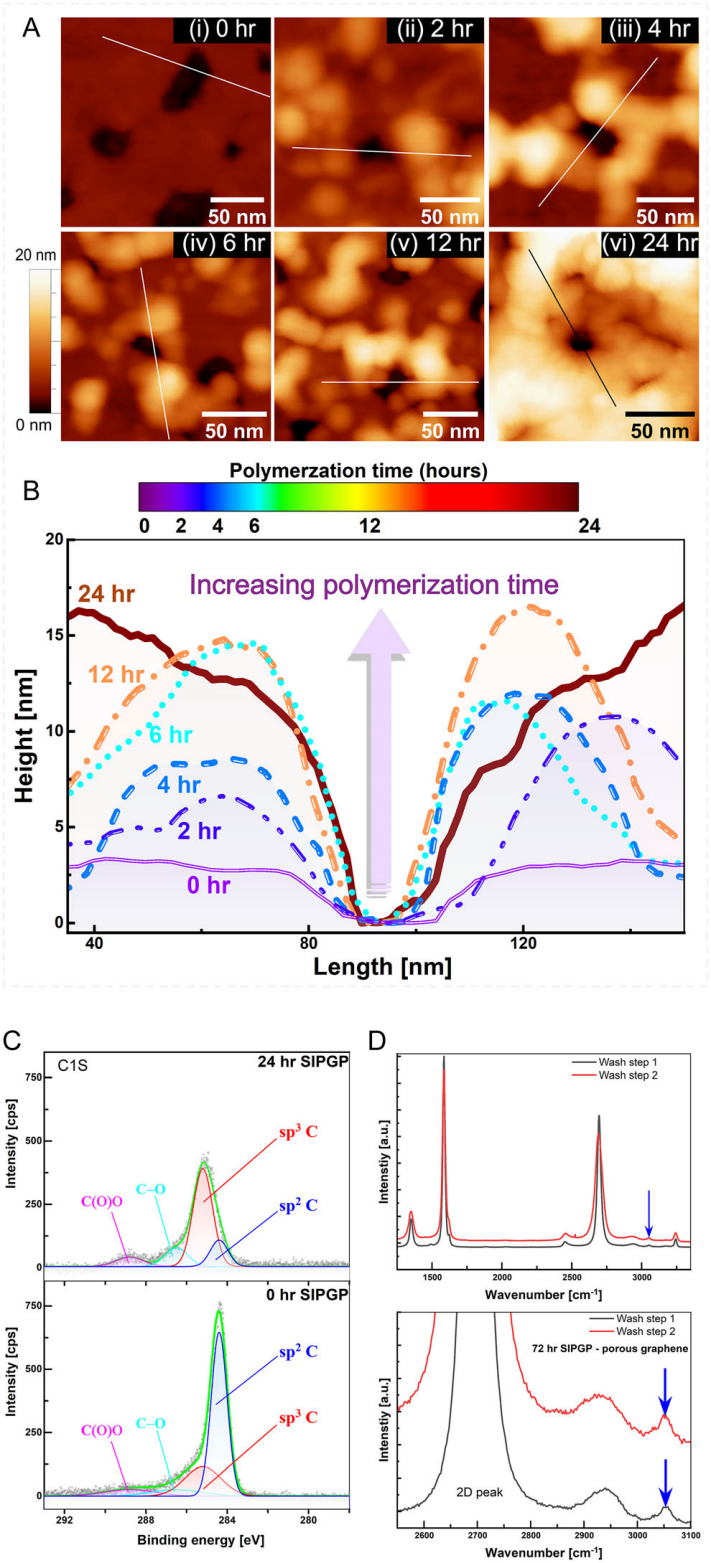
Influence of polymerization time on pore morphology. A) AFM images of selected regions of porous graphene at different stages of polymer grafting: (i) before, (ii) after 2 h, (iii) 4 h, (iv) 6 h, (v) 12 h, and (vi) 24 h of SIPGP with styrene monomers. The height variation range is 20 nm. B) AFM surface profiles of single pores after varying grafting times. The surface profile across the pore was cut out along the line shown in the AFM images (A (i) through (vi)). The surface profiles also represent the polymerization time using a color scale bar. C) Deconvoluted peaks of C 1*s* XPS spectra of films with 0 and 24 h of polymerization via SIPGP. The unpolymerized porous graphene sample revealed the dominant presence of *sp* ^2^ carbons (graphene) along with the presence of some *sp* ^3^ carbons from the defects on the basal plane and pore‐edges,^[^ ^44^ ^]^ together with fewer —CO, and —COOH groups possibly introduced during the graphene fabrication.^[^ ^33^, ^35^, ^44^, ^68^ ^]^ After 24 h of polymerization, the ratio between *sp* ^2^ and *sp* ^3^ carbons changed drastically indicating the substantially increased presence of polystyrene chains (the backbone contains *sp* ^3^ carbons). Notably, both —CO and —COOH groups remained to be present. Tentatively, these may be assigned to originate from the acid‐based etching step needed to remove the copper catalyst and liberating the graphene layer among other handling procedures.^[^ ^33^, ^35^, ^44^, ^68^ ^]^ D) Raman spectra of functionalized porous graphene samples after 72 h of SIPGP after washing with toluene. The blue arrows indicate the peak observed for polystyrene.

The morphologies of both modified and unmodified pores were investigated with a high‐resolution AFM. **Figure** **2**A shows the topographical changes of the porous graphene surface after growing polystyrene chains. All the samples were washed thoroughly by immersion and jet flow of various solvents (toluene, ethyl acetate, and ethanol) to remove physisorbed polymers and unreacted monomers before any characterization was done. Interestingly, the pores remained open even after functionalization. Moreover, the presence of a distinct “rim/halo” feature in the surface profiles of single pores confirms that the polymerization is initiated from the pore edges, consistent with the proposed model (some edge broadening is expected, see Figure S2, Supporting Information). The polymerization time has been varied systematically from 2 to 24 h to investigate the grafting process. The AFM images show that the unmodified graphene surface is very flat, with a 2–3 nm thickness. Prior publications on AFM analysis of graphene reported the height of a single layer of graphene to be ≈0.4 to 1 nm,^[^ ^71^, ^72^, ^73^ ^]^ the van der Waals distance being ≈0.34 nm, thus indicating the possible presence of both domains of few layers of the graphene on the surface (see Supporting Information) and single‐layer domains. Corresponding with our hypothesized model (Figure 1B) we can identify thicker domains in the magnitude of ≈6–10 nm after 2 h of polymerization. These regions and the amount visible at single pores, became consistently thicker with increasing polymerization time (Figure 2B). Additional characterization methods, such as X‐ray Photoelectron Spectroscopy (XPS) and Raman analysis (Figure 2C,D, see Figure S4, Supporting Information), were also carried out on the porous graphene films after the SIPGP process. The XPS data revealed a significant increase in the *sp* ^3^ carbons with extended polymerization time (24 h polymerization), pointing toward the presence of polystyrene (Figure 2C, Figure S5, Supporting Information). Raman analysis showed the presence of a polystyrene peak (aromatic C—H vibration at 3050 cm^−1^) only after an extended time of polymerization (72 h SIPGP) (please refer to Figure 2D, Figure S4, S15, Supporting Information). We assume that the low amounts of polystyrene in the relatively thin and scattered polymer domains drastically reduces their detection in many spectroscopic measurements like in FTIR. This is also consistent with earlier reports of the polystyrene peak in the Raman spectra showing 40× lower signal intensities compared to the 2D peak intensity of graphene layers.^[^ ^12^ ^]^ As a next step, we investigated the influence of the photo‐grafting process on the large‐scale morphology of the porous graphene.

**Figure 3:**
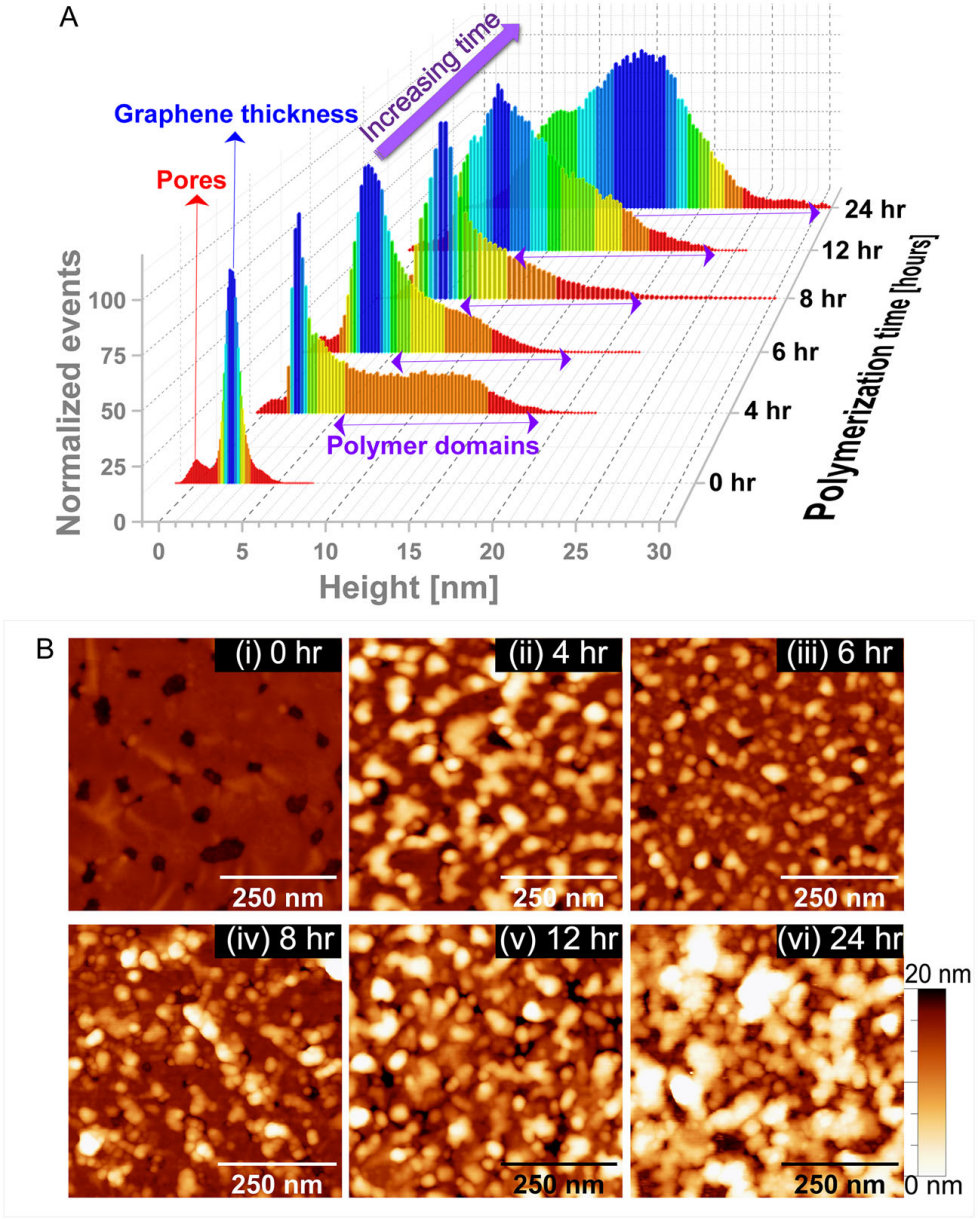
Large‐scale analysis of porous graphene layers with increasing polymerization time. A) Histogram from the AFM images (sampled area is more than 30 times the average pore diameter) measured after modified grafting times. The presence of domains with height > 8 nm increases with increasing polymerization time. The red arrow highlights the peak associated with the pore population, while the blue arrow highlights the peak related to the graphene basal plane. The violet double‐sided arrows show the presence of the distinct tail in the histogram related to the grafted polymer domains. B) Overview AFM images of porous graphene (i) before and after (ii) 4 h, (iii) 6 h, (iv) 8 h, (v) 12 h, and (vi) 24 h of SIPGP of styrene. All the scale bars are 250 nm, and the height range is 20 nm.

**Figure** **3** shows the progress of the polymer grafting on large‐scale topographic AFM micrographs of the porous graphene and the corresponding histogram analysis. Within 4 h of polymerization, we observed the presence of thicker domains predominantly along the pore edges while the graphene basal plane remained largely unaffected (dark brown background). After 6 h, however, a thickness change on the basal plane was noticeable (see Figure S3, Supporting Information). The histograms generated based on image analysis on a large scale further elucidate the polymerization process. The histogram of unmodified porous graphene shows a bimodal distribution, indicating the presence of pores (i.e., reference height = 0 nm) and the dominant peak assigned to the nonzero elevation of the basal plane (i.e., height ≈2 nm). The peak originating from the graphene basal plane in the pristine porous graphene shows a narrow distribution indicative of a very flat surface. Within 4 h of polymerization, we can still identify a graphene basal lamella peak at ≈2.3 nm. Moreover, we can observe a distinct tail of events in the histogram with heights of >10 nm. This is consistent with grafted polymer chains propagating from the pore edges toward the basal plane and is in line with previous observations, as we can clearly distinguish the basal plane from the modified pore edges. Furthermore, polymerization causes a significant broadening of the surface height distribution profile with the expected shift to higher values. This provides evidence of thicker polystyrene layers that increase with increased polymerization time. Interestingly, we also observe a shift in the peak observed for basal lamella, indicating that with extended polymerization time, the polymer chains’ growth and grafting process occur randomly. The histogram analysis of the AFM image obtained after 24 h of polymerization shows the presence of two distinct levels: 1) a level at 8 nm resulting from the thickening of the basal plane and 2) a peak at 15 nm thickness presumably from the polymers grafted along the pore edges.

**Figure 4:**
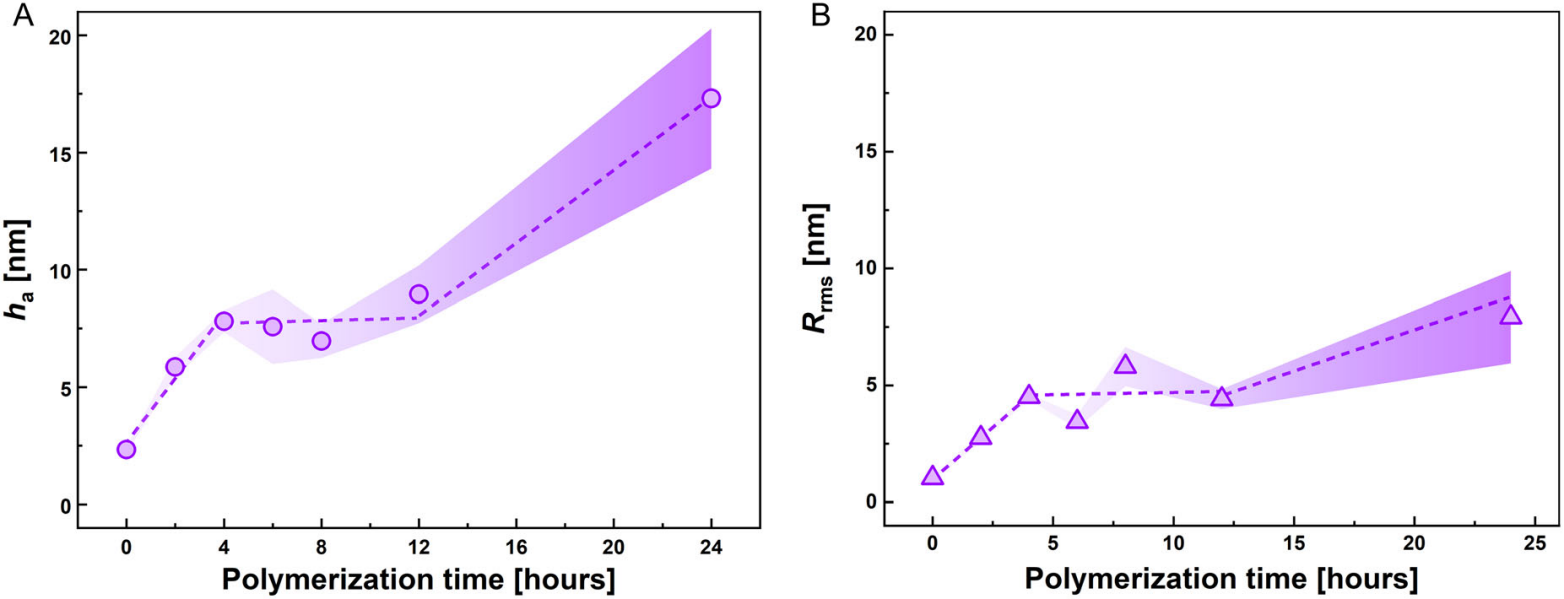
Evolution of average thickness and roughness of porous graphene layers with varying polymerization time. A) Influence of the polymerization time on average thickness (*h*_a_) of the porous graphene layer. B) Changes in the RMS roughness (*R*_rms_) of the porous graphene layer with increasing polymerization time.

Using the AFM images of functionalized and nonfunctionalized porous graphene, we calculated the average thickness (*h*_a_) and root mean square (RMS) roughness (*R*_rms_) to quantify the overall change of the porous graphene surface. We plotted *h*_a_ and *R*_rms_ as a function of polymerization time to quantitatively identify the kinetics of the polymerization process (see **Figure** **4**). We observed a seemingly linear increase in *h*_a_ and *R*_rms_ with increasing polymerization time until 4 h. Then, a pseudo‐plateau region emerged, after which *h*_a_ and *R*_rms_ increased again. Assuming a densely packed polymer layer grafted to isolated regions on the surface, in an ideal scenario, the average thickness and roughness of the film should increase linearly with the reaction time.^[^ ^74^, ^75^ ^]^ In the initial phase of polymer grafting, the length of an individual chain is expected to increase with increasing polymerization time. This behavior is evidenced by 1) the modified surface profile of the pores with increased polymerization time, 2) the evolution of the histogram analysis with increased reaction time, and finally 3) the linear increase in both *h*_a_ and *R*_rms_ with reaction time. The pseudo‐plateau region observed in the polymerized film might also be consistent with a progressive change in the conformation/orientation of the grafted polymers and the onset of polymer grafting from defectless areas of the graphene substrate.

Importantly, the thickening of the basal plane can be directly correlated to the onset of the pseudo‐plateau region. The onset of polymer grafting in the basal plane is also evident from the surface profile of the AFM images (Figure S3, Supporting Information), where we can observe a slight but noticeable thickening of the basal plane after 6 h of polymerization. Longer polymerization times lead to more randomly grafted polymers on the basal plane that might have a flat‐on/face‐on orientation. Consequently, the onset of the plateau may result in a smaller overall change in the thickness of the graphene layers as the polymer chains are added. This is likely because the grafting occurs across various areas, leading to only a subtle but noticeable change in the overall thickness.

**Figure 5:**
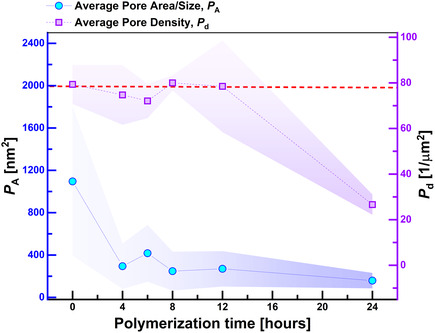
Influence of the polymerization time on average pore area/size and average pore density. The average pore size/area (*P*_A_) and the average pore density (*P*_d_) are plotted as a function of polymerization time. From the plot, a systematic decrease in the average pore area could be observed with the introduction of polystyrene along the pore edges while the pore density remained constant until 12 h of polymerization, after which we could observe an apparent decrease in both *P*_A_ and *P*_d_.

Unlike a UV‐ or plasma‐treated graphene surface, here the reactive sites for the grafting process are limited to defects introduced during the fabrication of the graphene. The reactive areas are assumed to be localized predominantly at the pore edges (Figure 1B) and include other minor scattered structural defects in the basal plane^[^ ^44^, ^45^ ^]^ and defects at the grain boundaries.^[^ ^76^ ^]^ Hence, the probability of grafting polymer is highest in a few areas on the porous graphene surface, with pore edges providing the most preferred sites, as evident from the AFM micrographs. Another effect of the grafting process along the pore edges was the influence of polymerization on the average pore size and pore density. As we expected, the polymers grafted along the pore edges should result in the presence of coiled polystyrene chains along the pores, which can, in turn, reduce the observed pore size. We may control the extent of pore size reduction by controlling the polymerization time. Hence, we quantified both average pore size/area (*P*_A_) and average pore density (*P*_d_) and plotted the values as a function of polymerization time (see **Figure** **5**). We observed a systematic decay of *P*_A_ (and pore diameter (*P*_Dia_), assuming for simplicity a circular pore $\left(P_{\text{Dia}\left(i\right)}=2\times \sqrt{\frac{P_{A\left(i\right)}}{\pi }}\right)$, see Figure S7, Supporting Information) with increasing polymerization time, while *P*_d_ remained almost constant until 12 h. Please refer to Figure S6, Supporting Information, for the details of the quantification process. Consistent with the thickness analysis, a significant decay of the *P*_d_ was also observed when the polymerization time was increased beyond 24 h. The abrupt increase in thickness of the polystyrene domains and the drastic reduction in the pore density clearly point toward a scattered grafting of polymer chains with partial clogging of pores after reaching a certain limit.

Nevertheless, the systematic decrease in pore diameter/pore sizes with lower polymerization time (0 h (*P*_A_ = 1095.3 ± 698.4 nm^2^) to 12 h (*P*_A_ = 269.7 ± 166.8 nm^2^)) strongly suggests the possibility of controlling the pore sizes of the porous graphene films. It is to be noted that the error bar is indicative of the variation of the pore sizes across the sample. The large standard deviation observed here is in line with the average variation of the sacrificial tungsten particles (average sizes = (1190.3 ± 831.8) nm^2^, Section S7, Supporting Information) present on the copper catalyst, which is directed by the random nature of the spinodal dewetting process of the tungsten film. Spinodal dewetting mechanisms can only be achieved within a limited range of tungsten films.^[^ ^56^, ^77^ ^]^ For example, when a thick film dewets, it may transition from a spinodal to a nucleation mechanism, resulting in low particle density.^[^ ^77^, ^78^ ^]^ Consequently, the number and sizes of the tungsten particles, as well as the pore sizes and density, are constrained due to the limitation of the thickness range of the tungsten film.^[^ ^77^, ^78^ ^]^ This limitation impacts the application potential of these porous graphene layers. Specific membrane applications, such as water desalination, require pore sizes on the sub‐nanometer scale to facilitate effective reverse or forward osmosis.^[^ ^79^, ^80^ ^]^ By attaching polymers at the pore edges, we can achieve from fourfold to sevenfold miniaturization of average pore sizes within 12 h (*P*_A_ = 269.7 ± 166.8 nm^2^) and 24 h (*P*_A_ = 158.2 ± 72.8 nm^2^) of polymerization, respectively. This offers a straightforward method for pore size fine‐tuning. Our work significantly broadens the applicability of these graphene layers in applications that demand pore sizes beyond what can be achieved by simply adjusting synthesis parameters.

The control over the pore sizes can therefore open additional application avenues for the work reported here, specifically for membranes that rely on size exclusion mechanisms (desalination is one such example)^[^ ^79^, ^80^ ^]^ or chemical selectivity. Moreover, the significance of this work lies in the selective grafting of polymers along the pore edges, especially at low polymerization times. The spatial selective modification of porous graphene, along with pore size control demonstrated in this work, represents a method for introducing functional polymers at the pore edges, which may be crucial for high‐performance membranes. Due to its ultra‐low mass, high mechanical strength, and excellent acoustic sensitivity, future applications of porous graphene may include membranes and diaphragms for acoustic ports (speakers and microphones) in mobile phones, acting as a pressure equalizer and water or dust protection (i.e., replacement of e‐PTFE or Si MEMS membranes).^[^ ^81^, ^82^ ^]^ Furthermore, the implementation of tunable porous graphene is foreseen to significantly contribute to gas separation for key technologies related to energy (e.g., hydrogen purification by removing CO_2_, CH_4_, and N_2_) and carbon capture (e.g., functionalized porous graphene membranes for selective CO_2_ chemisorption and release via pressure swing absorption). In these examples, the key advantage of porous graphene membranes lies in their ultrathin nature, leading to a very high permeance.^[^ ^83^, ^84^, ^85^, ^86^ ^]^ In summary, the selective pore edge modification will allow the fine tuning of pore sizes (selectivity via hydrodynamic radius), mechanical robustness (barrier), as well as the overall membrane mass (acoustic sensitivity). The addition of chemical functionality at the pore sites may improve chemo selectivity (e.g., amine for CO_2_ or chelators for ions).

**Figure 6:**
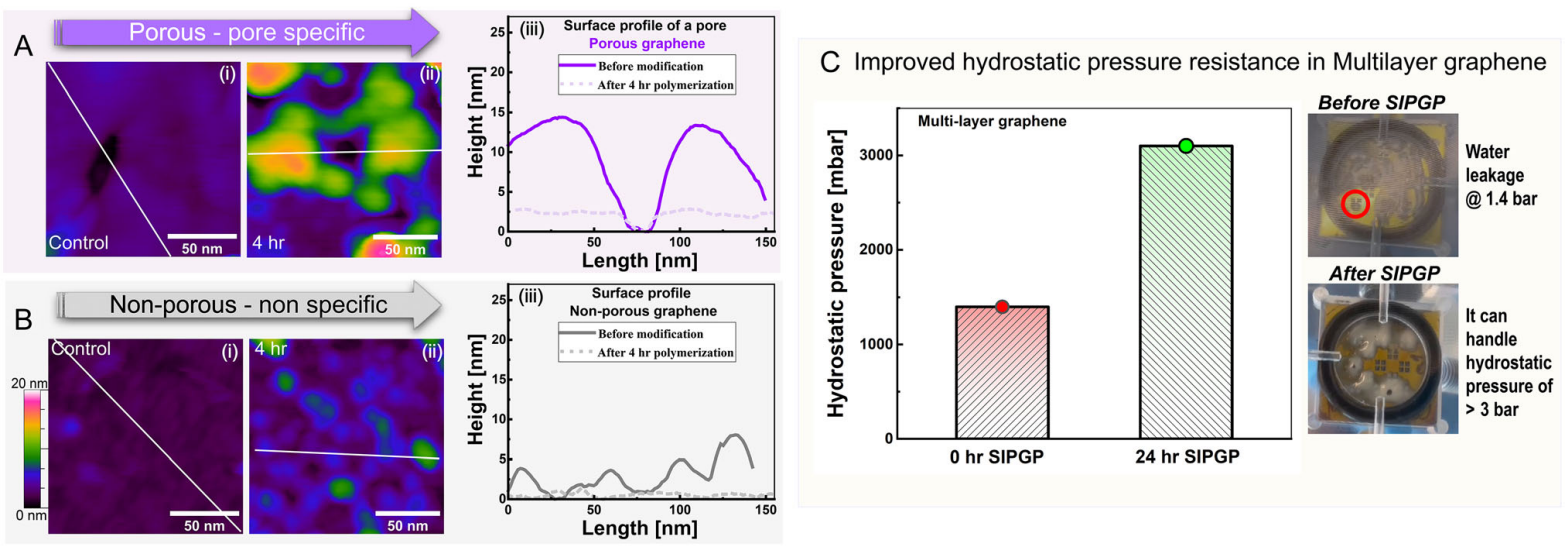
Comparison of the influence of the SIPGP process on porous versus nonporous graphene. A) High‐resolution AFM images of pores on porous graphene measured after (i) 0 h and (ii) 4 h of grafting polymerization. (iii) Surface profiles of the pores measured along the straight lines indicated in the AFM images (i)–(ii). B) Nonporous graphene measured after (i) 0 h and (ii) 4 h of grafting polymerization. (iii) Surface profiles of the basal plane of the graphene surface measured along the straight lines indicated in the AFM images (i)–(ii). Scale bars = 50 nm, and the height range = 20 nm. C) Influence of site‐specific polymerization on hydrostatic pressure. The procedure of hydrostatic pressure measurements is mentioned elsewhere and also described in Figure S19, Supporting Information.^[^ ^55^ ^]^ The measurements indicate increased hydrostatic pressure resistance (from 1.4 bar to 3 bar).

To further resolve the spatial specificity of the graphene modification process with SIPGP, we compared the pore morphology of porous graphene with the basal plane of the pristine graphene surface before and after the functionalization process. SIPGP was carried out with styrene monomer throughout this experimental series for 4 h. The AFM height profile demonstrates a clear distinction between the grafting zones of polystyrene in porous graphene as opposed to the pristine graphene without pores. In porous graphene, the grafted regions (green, yellow, and red areas in **Figure** **6**A) are concentrated mainly around the pores (black areas). In nonporous pristine graphene, the grafted regions have no spatial selectivity as the grafted polymers can be found randomly on the basal plane (blue areas in Figure 6B). The thickness of the grafted polymer also varies significantly in nonporous graphene compared to porous graphene. In porous graphene surfaces, however, thicker polymer domains can be observed primarily around the pores (surface profiles, Figure 6A). In nonporous graphene, owing to the randomness and scarcity of reactive sites (i.e., mainly abstractable hydrogen), the polymer domains are grafted indiscriminately and, to a lesser extent, nucleate on the basal plane (surface profile Figure 6B). This observation is consistent with previous studies, which report higher reactivity of the graphene edges compared to the basal plane.^[^ ^7^, ^45^, ^87^ ^]^ Interestingly, we observed similar enhanced edge reactivity for SIPGP when carried out on only partially grown graphene films exposing large edge domains. A pronounced thickening was observed due to polymer grafting at the graphene edges (please see Figure S8, Supporting Information, for further details). No preferred grafting region is present if pores are absent, like in pristine graphene. This comparison further elucidates the crucial role of predefined defects in achieving areal specificity during the functionalization of graphene surfaces.

The key feature of this work is the nanoscopic and site‐specific decoration of the pores with polymer rims. We achieved these decorations with a single step compared to the previously established methods of patterning, which require exhaustive preprocessing.^[^ ^12^, ^18^, ^30^, ^31^ ^]^ Additionally, the lateral resolution of grafted areas surpasses that of previously established methods of patterning through SIPGP by several orders of magnitude (Figure S18, Supporting Information).^[^ ^12^, ^18^, ^30^ ^]^ The presence of these nanoscale polymer domains around the pores can eventually mitigate defects associated with the processing of these materials.^[^ ^41^, ^42^, ^52^, ^53^ ^]^ Our preliminary results show an improved hydrostatic pressure resistance associated with site‐specific SIPGP (Figure 6C). Thus, site‐selective SIPGP provides a suitable pathway toward the fabrication of nanoporous graphene membranes for application areas such as waterproof membranes (as a replacement for e‐PTFE membranes) and water desalination, among others.^[^ ^79^, ^80^, ^88^ ^]^

## 3 Conclusion

This study establishes an efficient and scalable method for site‐selective functionalization of porous graphene films. Exploiting the enhanced reactivity of pore edges introduced during CVD graphene formation, these sites were selectively functionalized with polystyrene to nanoscale precision via a one‐step SIPGP method, avoiding any lithography or preprocessing step. AFM analysis confirmed polystyrene grafting preferentially along the pore edges within the graphene layer while the pores remained open. In addition, XPS and Raman spectroscopies confirmed the site‐specific grafting of styrene onto the porous graphene films. The histogram and surface profiling clearly show that thickness control over the grafted domains can be achieved by modifying the duration of the SIPGP process. Controlling the polymerization time also results in a varying degree of coverage around the pores, allowing for effective adjustment of average pore sizes. Up to sevenfold reduction of the pore sizes could be achieved by systematically varying the polymerization time, with average pore sizes ranging from (1095.3 ± 698.4) nm^2^ to (158.2 ± 72.8) nm^2^. The spatial specificity realized in this study enables the fabrication of polymer‐reinforced graphene pores, significantly improving the durability of porous graphene materials. Preliminary studies also demonstrate improved water column resistance when the porous graphene films are functionalized by this method, emphasizing its impact on the fabrication of graphene‐based waterproof membranes. This is particularly advantageous for onward research work for demanding membrane applications. Future research will focus on extending this work toward functional and robust graphene membranes, inspiring new avenues of exploration. Diverse polymer types with desired functionality (e.g., stimuli‐responsive polymers for controlling porosity on demand) and chemical functionalization (for improved selectivity) may also be grafted along the pores in a similar pathway, further, to improve the mechanical stability of the graphene membranes and for superior membrane performances concerning selectivity and adaptation to external stimuli. This work emphasizes the potential of selectively functionalizing porous graphene layers, which offers a valuable tool for fabricating the “ultimate” graphene‐based membrane.

## Conflict of Interest

The authors declare no conflict of interest.

## Supporting information

Supplementary Material

## Data Availability

The data that support the findings of this study are available from the corresponding author upon reasonable request.
