# Supplementary material for: Observations on Spatial Specificity in the Modification of Porous Graphene Layers
Source: ChemSusChem. 2025 Sep 19;18(21):e202501031. doi: 10.1002/cssc.202501031 (PMC12584971; doi:10.1002/cssc.202501031)
Supplement: Supplementary file 1 — Supplementary Material [file CSSC-18-e202501031-s001.pdf]

Supporting Information

Observations on Spatial Specificity in the Modification of Porous Graphene Layers

*Abhijna Das[†], Marcus Waser[†], Kyoungjun Choi, Theodor Bühler, Christelle Jablonski, Aaron H. Oechsle, Junggou Kwon, Murray Height, Thomas A. Jung, Renzo A. Raso**

A. Das, M. Waser, K. Choi, T. Bühler, C. Jablonski, J. Kwon, R. A. Raso
School of Life Sciences
Institute of Chemistry and Bioanalytics
University of Applied Sciences and Arts Northwestern Switzerland,
Hofackerstrasse 30, 4132 Muttenz, Switzerland (CH)
E-mail: renzo.raso@fhnw.ch

A. H. Oechsle, T. A. Jung
Laboratory for X-ray Nanoscience and Technologies, Center for Photon Science, Paul Scherrer Institute
Forschungsstrasse 111, 5232 Villigen, Switzerland (CH)

M. Height
HeiQ Materials AG
Rütistrasse 12, 8952 Schlieren, Switzerland (CH)

[†] These authors contributed equally to the work

Keywords: SIPGP, Porous graphene, Spatial specificity, Functionalization of graphene, Graphene edge reactivity

Contents

S1. Materials and Methods

- *Bottom-up porous graphene fabrication method*
- *Functionalization of porous graphene with polystyrene*
- *Transfer of graphene*

S2. Characterization methods

- *Atomic force microscopy*
- *Raman Spectroscopy*

S3. Changes in the AFM thickness of the basal plane

S4. Additional characterization of polymerized porous graphene films – Raman/XPS analysis

S5. Quantification of pore size/area, pore diameter, and pore density

S6. Enhanced reactivity at the domain edges of partially grown porous graphene films

S7. Reproducibility of the modification process in another type of porous graphene

- *Raman spectra analysis and SEM images of graphene prepared using two different types of copper substrate*
- *Influence of reaction time on pore morphology*
- *Influence of reaction time on large-scale porous graphene morphology*
- *Influence of reaction time on average thickness and rms roughness of the layer*

S8. The morphology of the layer when carried out on a pristine graphene layer without pores compared to porous graphene

- *Growth kinetics of SIPGP with styrene on non-porous graphene surfaces*
- *Comparison of grafted regions of polystyrene in both porous and non-porous films*

S9. Influence of UV light exposure and polymerization on the graphene

S10. Additional histogram analysis of large-scale area

S11. Additional pore surface profile analysis

S12. Comparison of photomasking/carbon deposition *vs* site specific SIPGP

S13. Hydrostatic pressure measurements

S1. Materials and Methods:

- **Bottom-up porous graphene fabrication method:**

To synthesize porous graphene layers, a W/Cu planar catalyst is required. A 4-nm-thick W film was deposited atop a bare Cu foil (JX Metals Corporation, 99.9% purity) using sputtering (FHR Pentaco 100). This Cu foil requires no further treatment. Sputtering was conducted at a pressure of 0.002 mbar, utilizing 100 standard cubic centimetres per minute (sccm) of Ar, and the 4-nm-thick W film was deposited for 30 seconds under 0.25 kW of DC plasma, along with 100 sccm of Ar. Furthermore, to mitigate surface roughness, a Cu foil (Alfa Aesar 46986, 99.8% purity) underwent Ar-ion beam treatment for 10 minutes. Samples made with as-received Cu-foil are referred to here as 'Type 1' while samples made with Cu foil with Ar-ion beam treatment are referred to as 'Type 2'. The sputtering process remained consistent with the aforementioned parameters. The chemical vapor deposition (CVD) synthesis of porous graphene is outlined elsewhere[1]. Briefly, the prepared W/Cu catalyst was housed within a Cu envelope (Alfa Aesar 46986) for further processing. Following the placement of the W/Cu catalyst in the Cu envelope positioned at the centre of a low-pressure chemical vapor deposition (CVD) furnace, the sample underwent annealing for 30 minutes at 950 °C with a flow of 50 sccm of H_2. During this annealing process, the 4-nm-thick W film underwent spinodal dewetting, transforming into circular-shaped W nanoparticles evenly distributed on the Cu surface. Subsequently, graphene synthesis was initiated at the same elevated temperature by introducing a mixture of 6 sccm of CH_4 and 50 sccm of H_2 at 800 mTorr for a duration of 15 minutes. The sample was then gradually cooled to room temperature while maintaining a flow of 100 sccm of H_2.

The non-porous graphene samples were synthesized on a copper catalyst (product number: GHZ-Z-H-V2) purchased from JX Metals. During the synthesis of pristine graphene, the Cu foil does not require any surface treatment before the process. The Cu foil was placed at the centre of the CVD furnace for the synthesis. Before initiating the graphene growth, the Cu foil was annealed for 20 minutes at 1000 °C with 50 sccm of H_2 flow. Then, graphene was grown on the copper catalyst at a temperature of 1000 °C by introducing a mixture of 50 sccm of CH_4 and 10 sccm of H_2 flow. The growth time was limited to 45 minutes. After the growth process, the sample was gradually cooled to room temperature in an environment with 20 sccm and 100 sccm of H_2 and Ar flow, respectively.

- **Functionalization of porous graphene with polystyrene:**

All reactions were carried out with the graphene still on the Cu foil catalyst as substrate. As graphene grows on both sides of the copper foil, the graphene on the backside was removed by floating the copper foil with the backside on an aqueous 0.5M solution of $(NH_4)_2S_2O_8$ for 10 minutes. The copper foil was then placed with the backside on a paper tissue and the backside was slid off by pulling the foil over the tissue. The copper foil with the remaining graphene on the upper side was rinsed with deionized water and air dried. It was then used for polymerisation reactions. Monomers were inhibitor free, and oxygen was removed by applying two freeze-thaw cycles. The graphene sample was immersed in the styrene monomer under argon

atmosphere for the polymerization reaction. Irradiation with UV fluorescent lamp with a spectral distribution between 300 and 400 nm (intensity maximum at λ = 365 nm with a total power of 1 mW/cm^2). It is to be noted that the power of the UV radiation is 5 times less than what is typically reported on the SIPGP of graphene[2]. After UV irradiation, the samples were removed and thoroughly washed with toluene, ethyl acetate, and ethanol to remove physisorbed polymer and unreacted monomers.

- **Transfer of graphene:**

To remove the polymer functionalized CVD graphene from the copper catalyst substrate, the copper foil was etched off for 3 hours by floating the sample, with the copper side facing the aqueous surface, with a concentration of 0.5M $(NH_4)_2S_2O_8$. Furthermore, the $(NH_4)_2S_2O_8$ solution also assists in dissolving the W droplets present on the copper surface. Hence, during the etching process with $(NH_4)_2S_2O_8$ solution, W droplets are removed. After the total dissolution of the Cu foil and parallel removal of the W droplets, the functionalized graphene sheet remained floating on the $(NH_4)_2S_2O_8$ solution and could be observed by eye. At this stage it is possible that reactive hydroxyl radicals formed in presence of APS in water, may react with the graphene leading to functionalities such as hydroxyls, aldehydes, epoxides, carboxylic acids.[3,4] The functionalized graphene sheet was then taken up by using a SiO_2/Si wafer and transferred to water and allowed to float for 1h. It was then picked up on a Si wafer with a 300 nm SiO_2 layer. The polymerized pristine graphene samples were also transferred to a Si wafer using the same process as described here. Unlike the polymerized samples, a thicker film of poly(methyl methacrylate) (PMMA) was spin-coated on the pristine graphene before transferring it from the copper catalyst using the outlined etching process. For unpolymerized single-layer graphene, the PMMA layer acts as a supporting layer, which prevents it from disintegrating during the transfer process. After transferring the graphene onto a Si wafer, the PMMA layer was removed from the graphene by immersing the sample in an acetone bath for 1 hour.

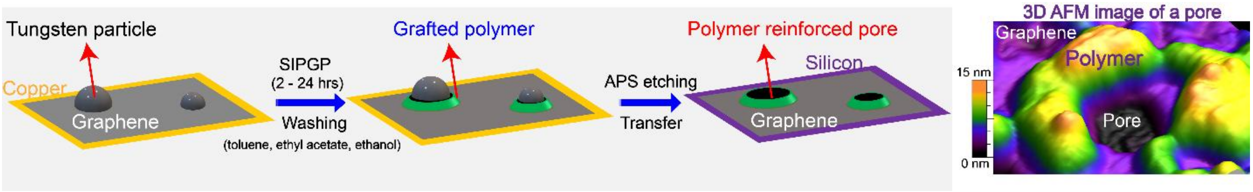

Figure S1. Schematic diagram of the SIPGP process with styrene as a monomer on porous graphene films. A 3D AFM image of a polymerized pore is also shown in the figure.

S2. Characterization methods:

- **Atomic Force Microscopy (AFM):**

We have chosen atomic force microscopy as the primary characterization technique in this study since it provides information about the morphology and the thickness evolution of the samples with reaction time in a non-destructive way. We have used a Bruker Multimode 8-HR atomic force microscope to obtain the AFM images. All the images were obtained in tapping mode with Bruker RTESPA-150 AFM tips. The

resonance frequency of the tips varied from 125 – 155 kHz and the nominal radius of curvature of the tips was 8 nm. The features measured in this study were much larger than 8 nm, however, some edge broadening effects (*ca.* 10 nm) should be considered, especially at the pore edges, due to finite radius of curvature of the tip (see Figure S2)[5]. The images were processed with WSxM software[6]. For height and the roughness analysis, measured data was collected from at least three different regions of the samples. Images of sizes ranging exclusively from (500 × 500) nm^2 and (3,000 × 3,000) nm^2 were used to calculate the average height (h_a) and roughness (R_{rms}) of both modified and unmodified films to account for the sub-micron size (< 100nm) pores of the graphene films. The error bars in h_a and R_{rms} indicate the heterogeneity of the polymer domains observed in the graphene films.

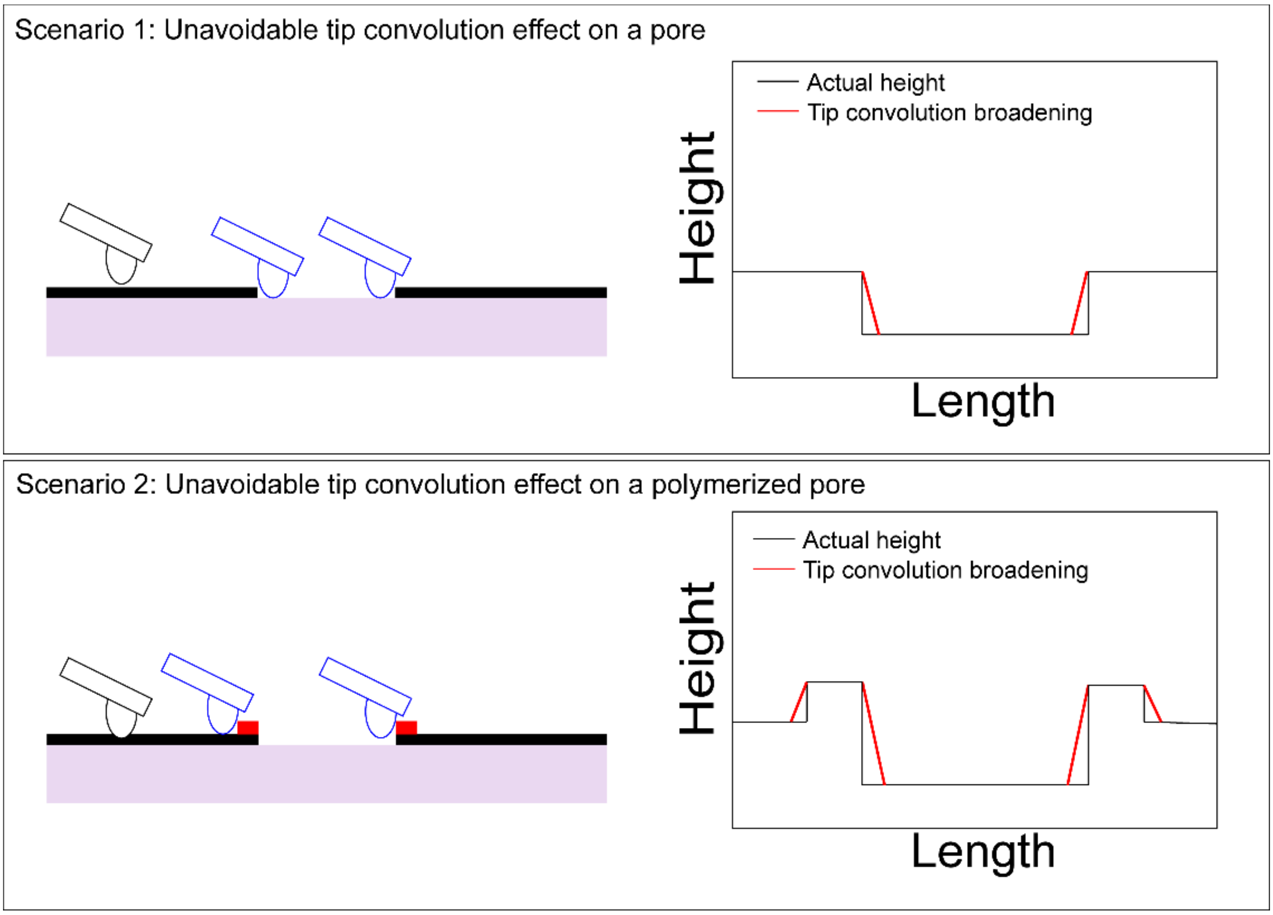

Figure S2. Schematic diagram depicting the radius of curvature induced edge broadening during AFM measurements.

- **Raman Spectroscopy:**

Raman spectra were recorded with a confocal Raman microscope (WITec alpha 500/300R+ ; WITec GmbH, Ulm, Germany) using implemented Zeiss objectives (100× NA = 0.95). The excitation wavelength of the Nd:YAG laser was 532 nm actuated at 5 and 10 mW. Signals were detected by a back-illuminated CCD camera after passing a 50-µm pinhole. The spectral resolution was 4 cm^{-1}. Integration time for each Raman spectrum acquisition pixel was 0.2 s. The collected Raman spectra were processed using the software

WITec Project Plus (WITec GmbH, Ulm, Germany) to remove Cosmic ray occurrences, and to subtract the background.

- **X-ray photoelectron spectroscopy:**

The X-ray photoelectron spectroscopy (XPS) analysis was performed at the Laboratory for X-ray Nanoscience and Technologies at Paul Scherrer Institute PSI. The porous graphene samples transferred on silicon oxide substrate (0 hr polymerization and 24 hr polymerization) were used for the spectroscopic analysis. The spectra reported here were averaged from 4 scans with 10 eV of pass energy. The step size for each measurement was 0.01 eV, and the dwell time was 0.2 s. The spectra (deconvolution and background subtraction) were analysed using OriginPro software. Before the deconvolution process, the background was subtracted (using a standard Shirley method).

S3. Changes in the AFM thickness of the basal plane:

To demonstrate the changes in thickness in the basal plane with increasing polymerization time, we used two separate surface profiles of the AFM images obtained at varying reaction times. The unmodified few layers of porous graphene showed a basal plane thickness of ca. 2.3 nm – 3.0 nm, similar to the histogram peak we observed from the histogram analysis. With 4 hrs of polymerization, we could still identify the basal plane at 2.3 nm – 3.0 nm. Additionally, we observed thick domains with heights of > 10 nm, especially around the graphene pore edges. Further polymerization (6 hrs) led to a slight increase in the basal plane thickness (Figure S3. iii(a-b)). Interestingly, polymerization times of 12 hrs and 24 hrs led to significant increases in the basal plane (of > 5 nm in both cases, Figure S3. iv(a-b) and Figure S3. v(a-b)) indicating spatial growth of the polymers with time.

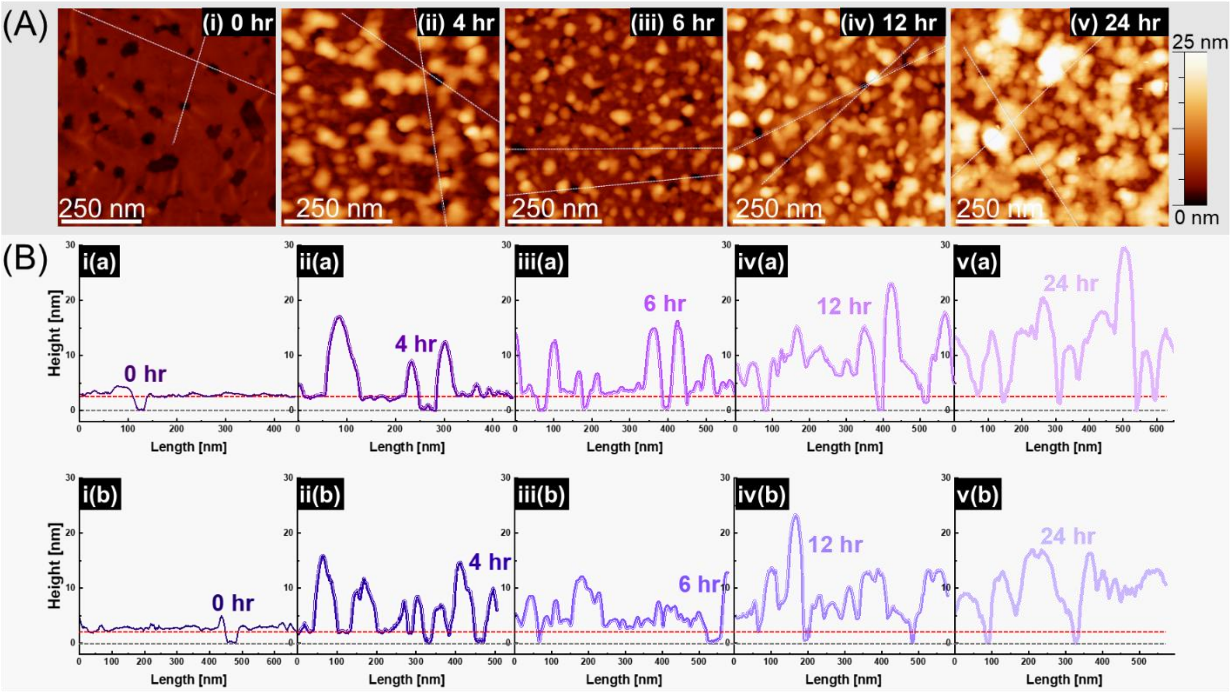

Figure S3. (A) Overview AFM images of single layer/few layers porous graphene (i) before and after (ii) 4 hr (iii) 6 hr, (iv) 12 hr, and (iv) 24 hr of SIPGP with styrene monomer. All the scale bars are 250 nm and the height range is 25 nm. (B) Surface profiles of two different areas (a, b) obtained from the images i-v showing evolution of height of the basal plane as well as the pore edges with increasing polymerization time.

S4. Additional characterization of polymerized porous graphene films – Raman/XPS analysis

Unlike other works on SIPGP, where monolithic polymer carpets were grown on graphene films,[2,7] this work deals with scattered polymer domains around the pores. Which essentially implies that fewer polymer domains are present on the graphene film, leading to the presence of a relatively heterogeneous polymer layer. Hence, the sensitivity of the detection of the polymer through techniques such as Raman and FTIR also reduces drastically. As the study by Steenackers and colleagues[2] points out, the signal for polystyrene in the Raman spectra (peak at 3050 cm^{-1}) was 40x less than the 2D peak intensity.[2] In this scenario, where the accumulation of polymers was much less than in prior reports, we assume the polystyrene peak was below the detection limit. Therefore, high-resolution measurements *via* atomic force microscopy were preferred as the primary characterization method, showing the presence of polymers along the pore edges. Additionally, the SIPGP process on porous graphene was carried out for 72 hours to grow a homogeneous polymer carpet along the basal plane. The sample was washed several times by immersing it in a toluene bath and by a flow of toluene to remove undesired physisorbed polymers. After each subsequent washing step, we observed a slight but noticeable polystyrene peak at 3050 cm^{-1}. While in both cases, we could detect the polystyrene signal, however, it was approximately 50x less than the 2D peak intensity (Figure S4 (A)). The thickness of the washed layer was also investigated *via* AFM, which showed a thickness of *ca.*

25 nm on the graphene film (Figure S4(B)). Hence, we can confirm that polystyrene was attached to the graphene films.

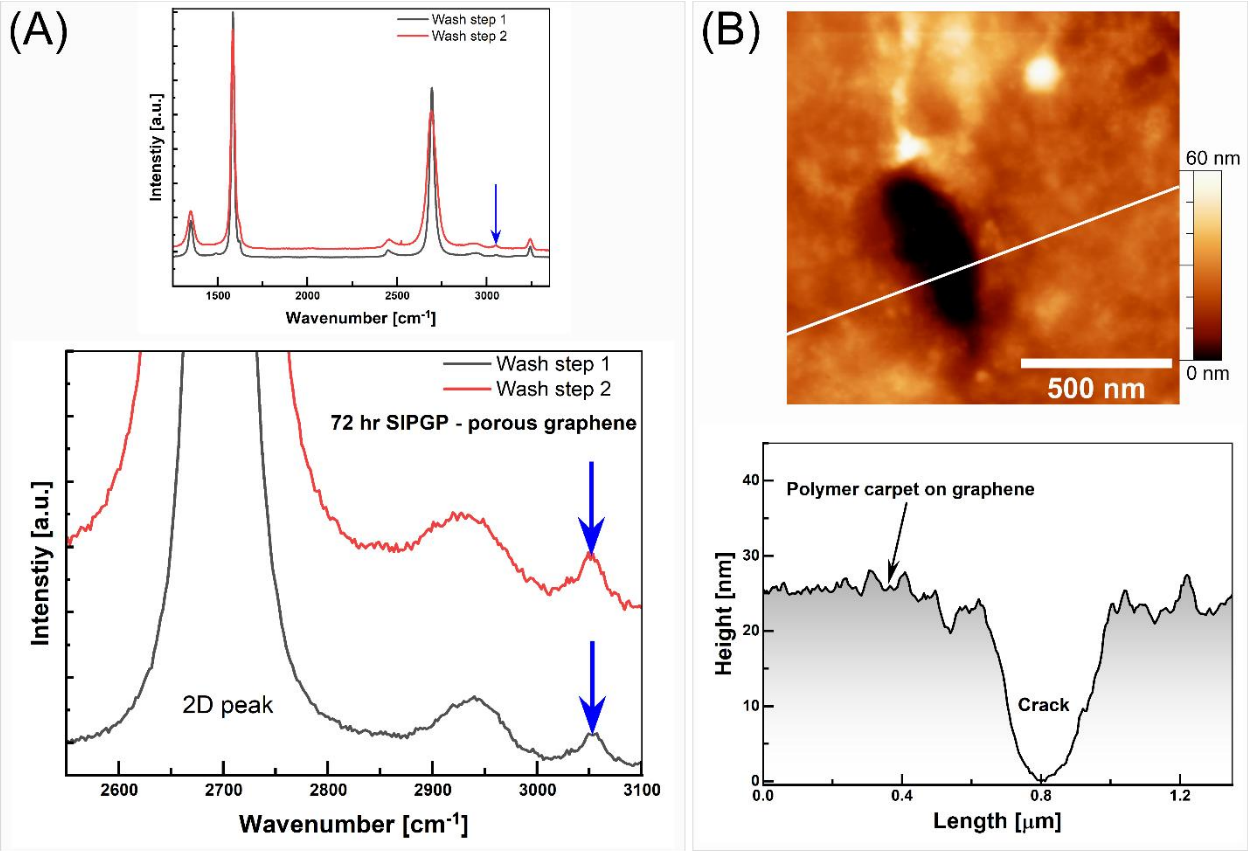

Figure S4. (A) Raman spectra of polymerized porous graphene samples (72 hours of SIPGP) after washing with toluene. The blue arrows indicate the peak observed for polystyrene. The intensity of the polystyrene peaks observed here were approximately 50 x less than the 2D peak intensities, highlighting the detection limit of the characterization techniques such as Raman. (B) The AFM image of the porous graphene samples (72 hours of SIPGP) after washing with toluene. The measurement was carried out in an area where a crack was visible to measure the thickness of the grown polymer layer. The surface profile along the crack reveals the thickness of the polymer layer to be ca. 25 nm. The scale bar of the image is 500 nm, and the height range is 60 nm.

Furthermore, we also carried out additional characterization with XPS (C1S), which might be more surface-sensitive. The XPS measurements were carried out on the porous graphene films before and after polymerization *via* SIPGP (24 hours). The graphene film without any functionalization showed the presence of a large peak associated with sp^2 carbon at ca. (284.4 ± 0.1) eV.[8] Additionally, the presence of sp^3 carbon (285.2 ± 0.1) eV[8], small but noticeable peaks associated with CO groups (286.5 ± 0.1) eV[9] and COOH groups (288.8 ± 0.1) eV[9] were also observed, possibly resulting from the defect areas in the graphene film.[3,10] Interestingly, a very noticeable shift in the overall spectra was observed with 24-hour polymerization time. Deconvolution of the spectrum revealed a significant increase in the presence of sp^3 carbons, indicating the presence of polystyrene (resulting from the backbone specifically).

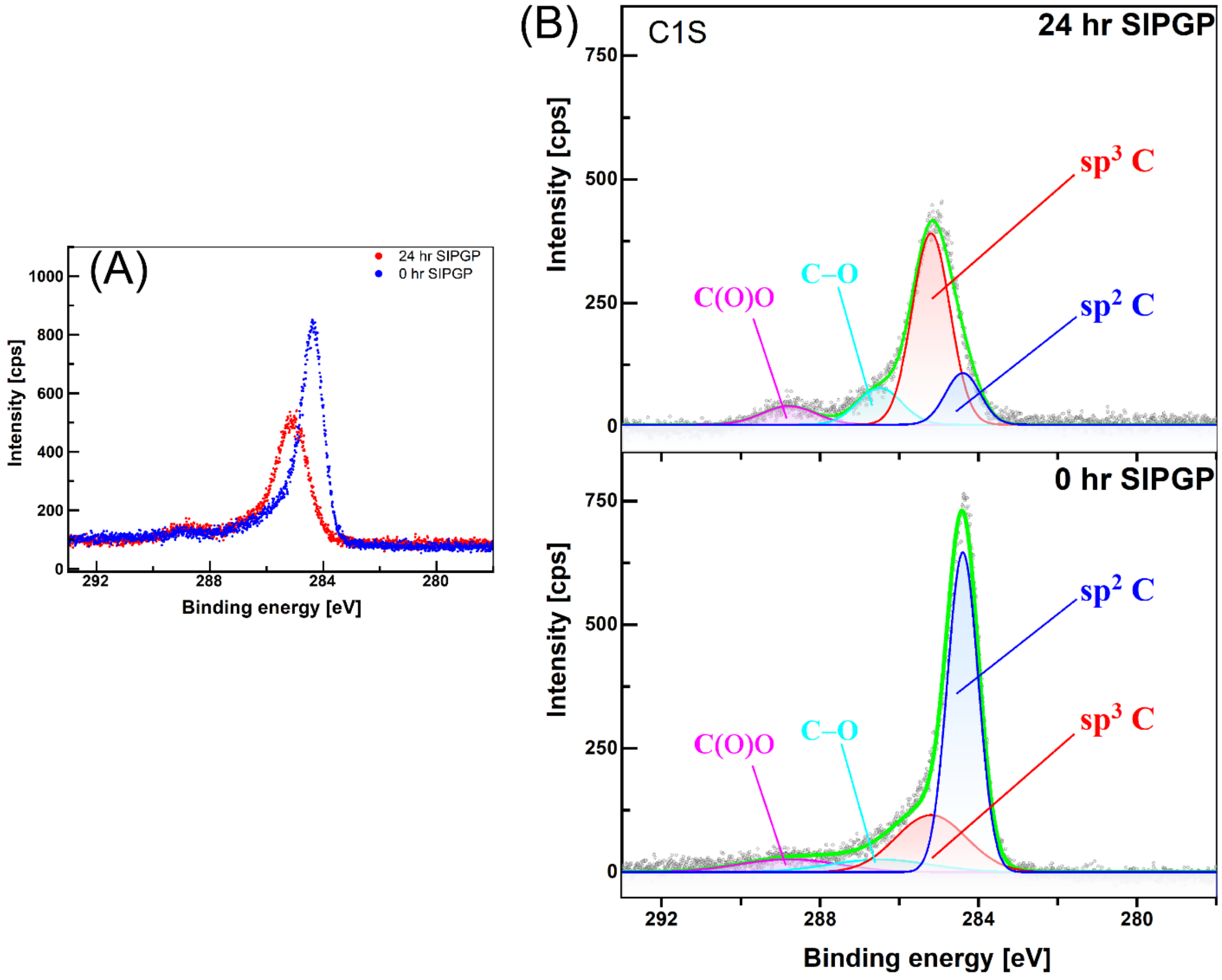

Figure S5. (A) XPS spectrum of porous graphene films before (blue) and after (red) polymerization *via* SIPGP for 24 hours. (B) Deconvoluted peaks of C1S XPS spectra of films with 0 hr and 24 hr of polymerization *via* SIPGP. The unpolymerized porous graphene sample revealed the dominant presence of sp^2 carbons (graphene) along with the presence of sp^3 carbons (defects on the basal plane, and edges),[3] -CO, and -COOH groups (possibly introduced during their processing steps).[3,10–12] After 24 hours of polymerization, the ratio between sp^2 and sp^3 carbons changed drastically indicating the presence of polystyrene chains (the back-bone contains sp^3 carbons). Additionally, both -CO and -COOH groups were also present on the sample, both groups could be introduced on the sample during the acid-based etching step from the copper catalyst and handling processes.[3,10–12]

S5. Quantification of average pore size/area, pore diameter, and pore density

We have used ImageJ software to quantify both the average pore size/area and pore density from the AFM images obtained for porous graphene films after varying polymerization times. After converting the images to 8-bit RGB, the pixel population originating from the pores, the basal plane, and the polymerized areas were distinguished using histogram profiles obtained from the images. Using a simple image segregation process, the images were converted to binary images separating the other populations from the pores. The "analyze particle function" was used to quantify the pores assuming a circularity range of 0-1, and a minimum pore size of 25 nm^2. The average pore size was calculated by averaging the areas of all the measured pores. The average pore density was calculated by dividing the total pore number with the total size of the image used to calculate the data. At least 3 AFM images obtained from different regions of the samples were used to measure the data to account for the possible heterogeneity of the layer. The average pore

diameter was calculated from the average pore area assuming circular pores. The average pore diameter was determined from the pore sizes by employing the following equation, followed by the identical averaging process.

$$P_{Dia(i)} = 2 \times \sqrt{\frac{P_{A(i)}}{\pi}})$$

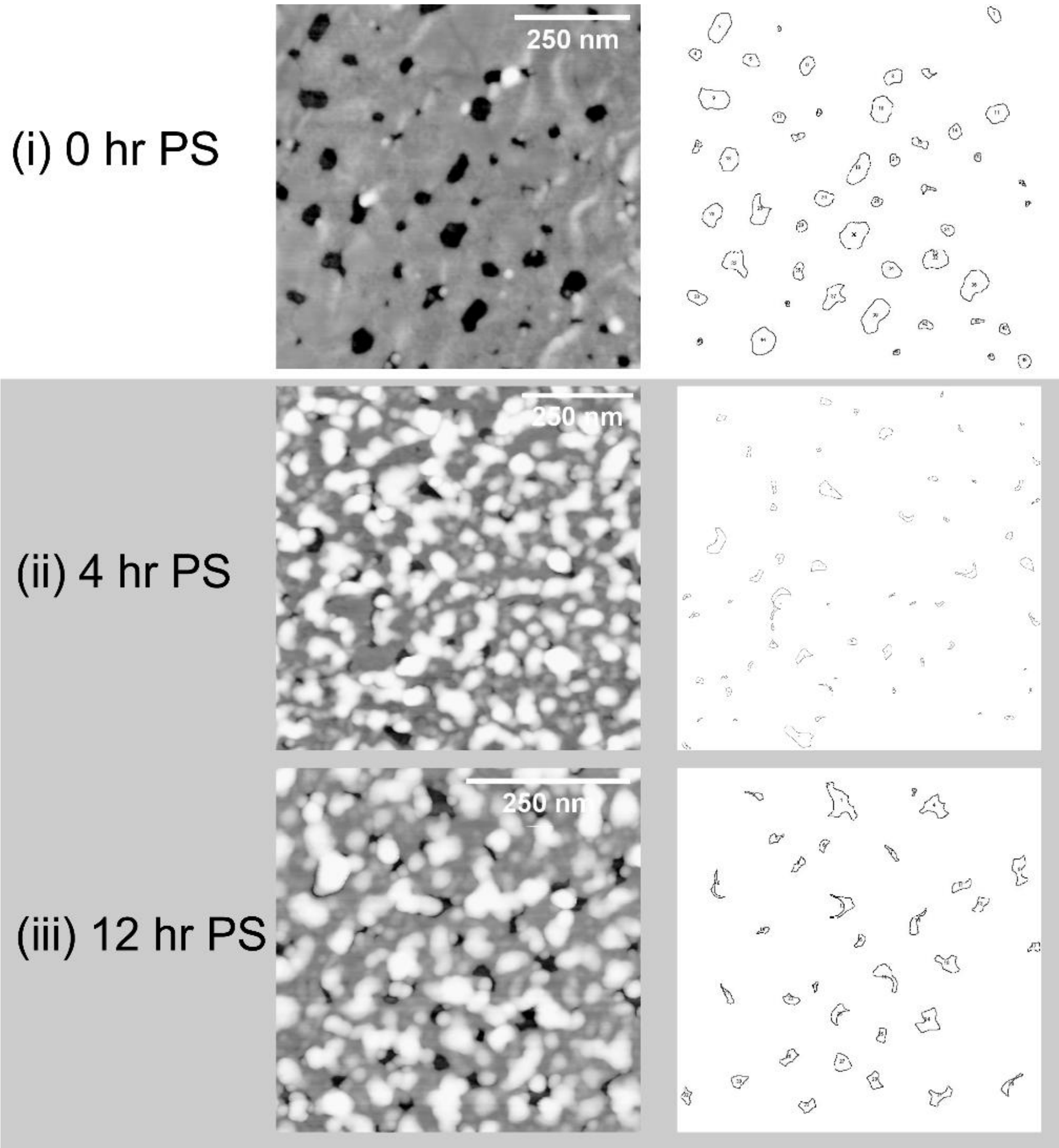

Figure S6. The quantification of pore size/area and pore count using ImageJ for porous graphene films polymerized for (i) 0 hours, (ii) 4 hrs, and (iii) 12 hrs. The left column shows the images after converting to the RGB scale, and the right column shows the outlines of the measured pores, which are consistent with the observed pores in the AFM images. The scale bar of the images is 250 nm.

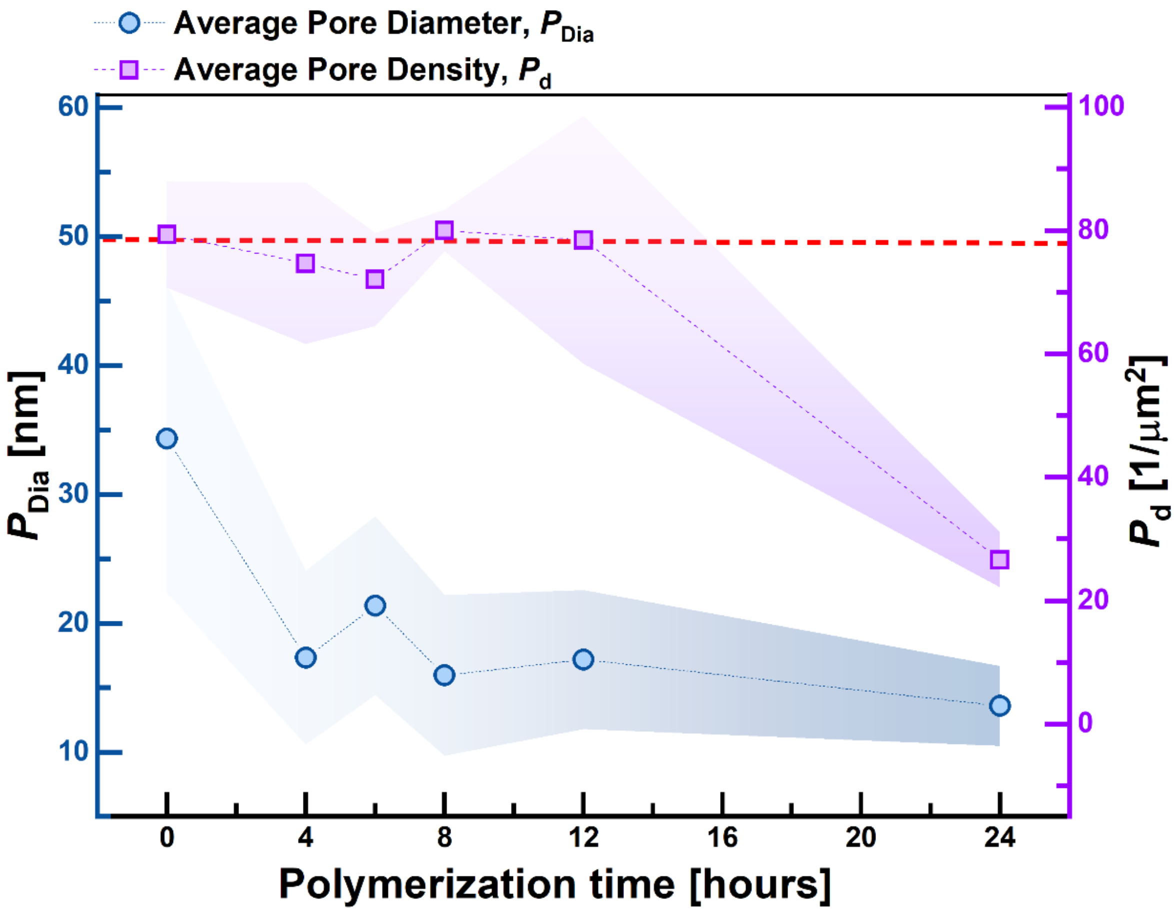

Figure S7. Influence of the polymerization time on average pore diameter and average pore density. The average pore diameter (P_{Dia}) and the average pore density (P_d) plotted as a function of polymerization time. The pore diameter was calculated by assuming the pore shape as circular, and the following equation was used to calculate the pore diameter:

$$P_{Dia(i)} = 2 \times \sqrt{\frac{P_{A(i)}}{\pi}}$$

where $P_{Dia(i)}$ is the diameter of an individual pore and $P_{A(i)}$ is the area/size of an individual pore. From the plot, systematic decrease in the average pore area could be observed with the introduction of polystyrene along the pore edges while the pore density remained constant until 12 hours of polymerization, after which we could observe a clear decrease in both P_{Dia} and P_d.

S6. Enhanced reactivity at the domain edges of partially grown porous graphene films

Graphene edges have been reported to have been more reactive than the basal plane due to the presence of dangling bonds[4,13,14] and one can expect to have similar dangling bonds which are hydrogenated at the pore edges too. Hence, we expect better reactivity at the pore edges compared to the basal plane. We conducted the SIPGP polymerization on partially grown porous graphene film (films where nucleated domains of epitaxial graphene films are not merged to form a continuous layer) to demonstrate edge specific SIPGP of polystyrene We could clearly observe edge thickening of polymers along the domain edges. These domain edges, which reflect similar reactivity as graphene edges, would be highly reactive for the grafting process of the polystyrene chains. Hence, the thickening of the grafted polymer along the domain edges clearly reflects its enhanced reactivity and presence of abstractable hydrogen, aligning with the findings of this study.

Figure S8 shows the AFM images of the areas of graphene obtained after the SIPGP process. Interesting from the AFM images and the subsequent height profiles, we could clearly observe significant thickened edges of each domain of graphene (brighter areas in the AFM images are present along each domain edge). The thickened graphene domain edge suggests an enhanced reactivity of the graphene edges compared to the basal plane which is consistent to the mechanistic model of higher reactivity along graphene edges/defects presented in this study.

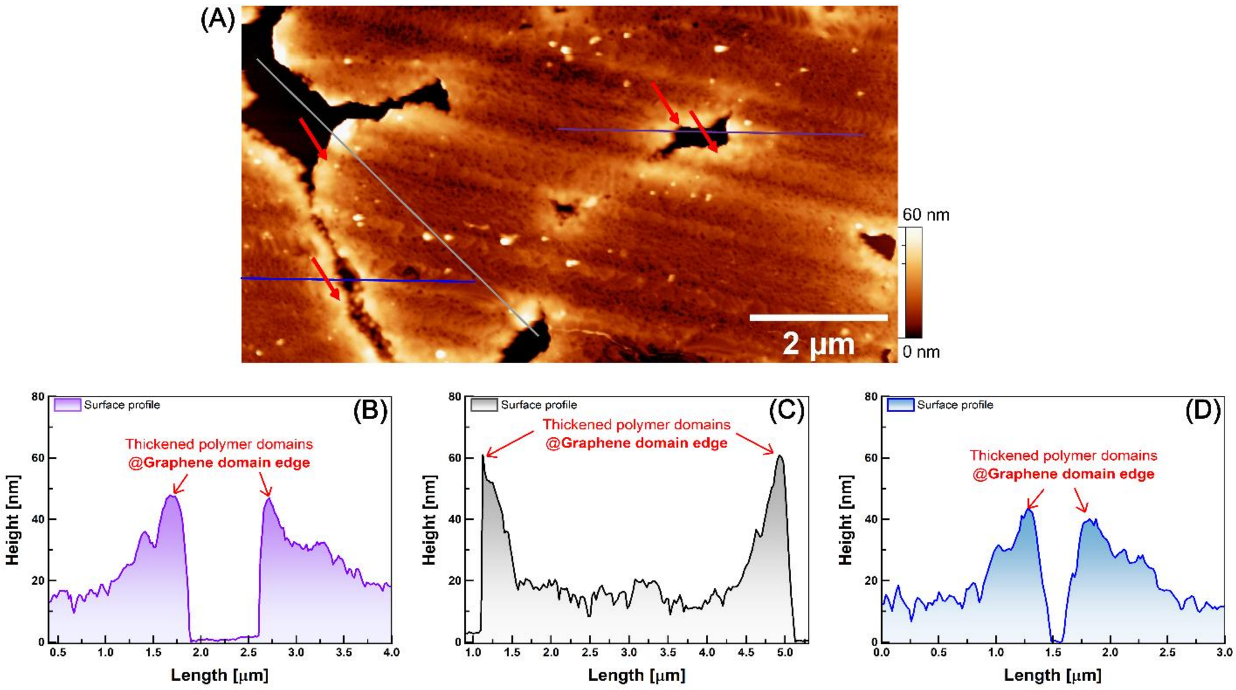

Figure S8. Influence of polymerization on graphene edges. (A) Large scale AFM image of a partially grown porous graphene film that was polymerized *via* SIPGP for 24 hours using styrene. Bright areas in the AFM image represent thicker domains of polymers which are clearly visible along all the partially grown graphene domain edges (highlighted by the red arrows). The scale bar is 2 μm and the height ranges from 0 nm to 60 nm. (B-C) The surface profiles obtained from the image (A) along the lines drawn (violet, black, and blue) in the images which show thickened polymer domains along the graphene domain edges highlighting higher reactivity along the graphene edges.

S7. Reproducibility of the modification process in another type of porous graphene:

- **Raman spectra analysis and SEM images of graphene prepared using two different types of copper substrate:**

To further investigate the feasibility of the process, we tested the influence of polymerization on another type of porous graphene. The porous graphene was grown in another type of copper substrate with a rougher surface structure (Figure S9B). The process is outlined in the materials and methods section in detail. Figure S9 shows the comparison of the morphology of graphene on types of copper substrates and the comparison of Raman spectra between the two samples. We calculated I_D/I_G and $I_D/I_{D'}$ ratios from the Raman spectra measured from the single layer domains of the two types of porous graphene as the ratios can help identify both the presence and the origin of defects, respectively. The I_D/I_G ratios were 0.364 for type 1 porous graphene, 0.427 for type 2 porous graphene, and 0.069 for non-porous graphene, with the $I_D/I_{D'}$ ratios being

3.309 for type 1 porous graphene and 3.347 for type 2 porous graphene. The notable increase in the I_D/I_G ratios observed for porous graphene samples suggests an increased presence of defects on porous graphene surfaces compared to graphene surfaces without pores. In addition, $I_D/I_{D'}$ ratios for both graphene types were close to 3, indicating that the defects are due to disordered edges[1,15,16] rather than the presence of vacancy-type defects or sp^3 hybridized carbons.[1,15,16] While the Raman spectra are comparable in both cases, the difference in their morphology comes from the observed pore density and the pore sizes. The tungsten particles, formed during the dewetting process of the deposited tungsten film, locally inhibit graphene growth, with pores forming at the areas where the tungsten particles were present. Hence, the sizes and density of the W particles directly reflects both the pore sizes and the pore density. The porous graphene used earlier (type 1, average sizes = (1190.3 ± 831.8) nm^2) shows larger tungsten particles than the other porous graphene type (type 2, average sizes = (723.8 ± 602.1) nm^2). Reciprocally, pore density seems to be higher in porous graphene type 2 compared to porous graphene type 1. Nevertheless, as the I_D/I_G ratios are comparable in both cases, we expect similar polymer growth kinetics in both graphene surfaces where the polymer should be grafted densely around the pore edges.

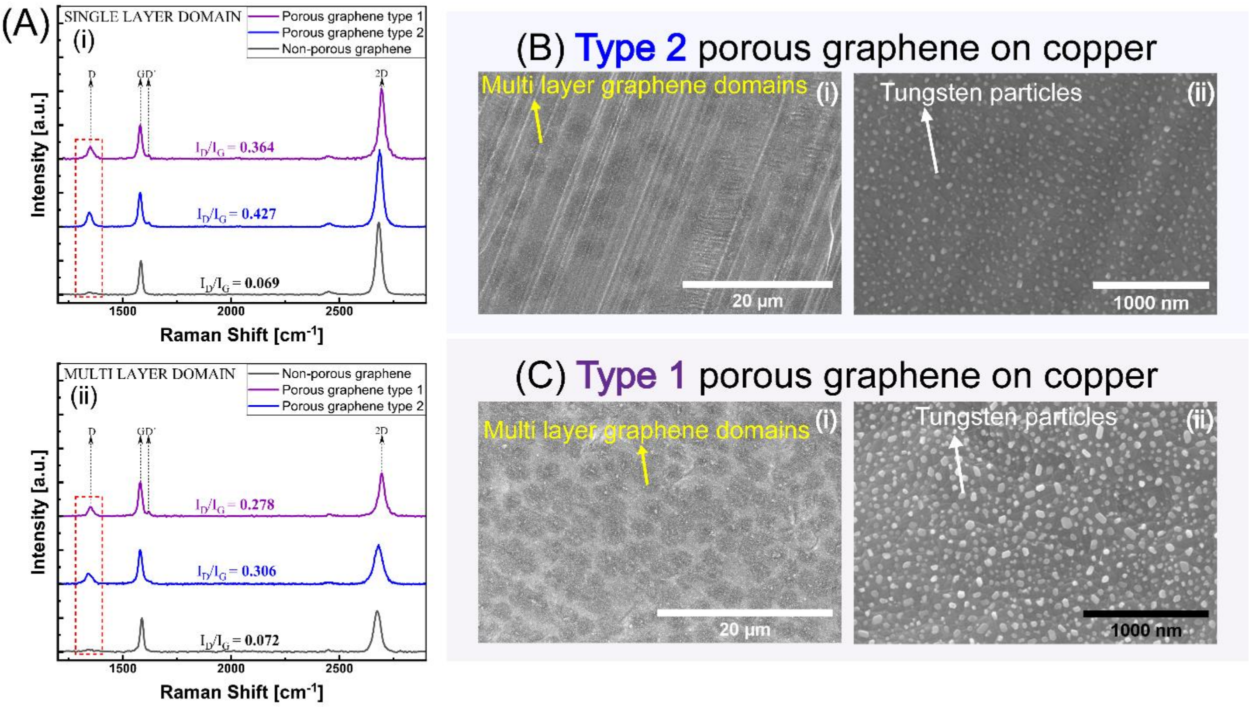

Figure S9. (A) Raman spectra obtained for pristine graphene (black), porous graphene type 1 (violet), and porous graphene type 2 (blue). The I_D/I_G ratios clearly indicate the changes in the fractions of defects in the graphene with the introduction of pores for (i) single layer domains and (ii) multi-layer domains. (B) SEM images (i) large-scale (scale bar = 20 µm), and (ii) small-scale (scale bar = 1 µm) showing the morphology of the copper where porous graphene type 2 is grown with CVD. The multi-layer domains of graphene are clearly visible in the SEM images. Furthermore, the tungsten particles formed during the dewetting process which inhibit the growth of graphene is also visible in the SEM images. (C) SEM images (i) large scale (scale bar = 20 µm), and (ii) small scale (scale bar = 1 µm) showing the morphology of the copper where porous graphene type 1 is grown with CVD. The multi-layer domains of graphene (in the large-scale image) and the tungsten particles (small-scale image) are clearly visible in the SEM images.

- **Influence of reaction time on pore morphology:**

Polymerization of the graphene films was conducted for times between 2 – 8 hrs to investigate the influence of polymerization on this graphene film. Figure S4 shows the evolution of the morphology of the graphene pore edges with increasing reaction time. From the surface profiles, we can clearly observe a thickening of pore edges that can be associated with the increasing reaction time. Consistent with the type 1 porous graphene, grafted polymer domains can be identified primarily along the pore edges in this type of graphene as well and the basal plane shows minimal presence of polymer domains compared to pore edges.

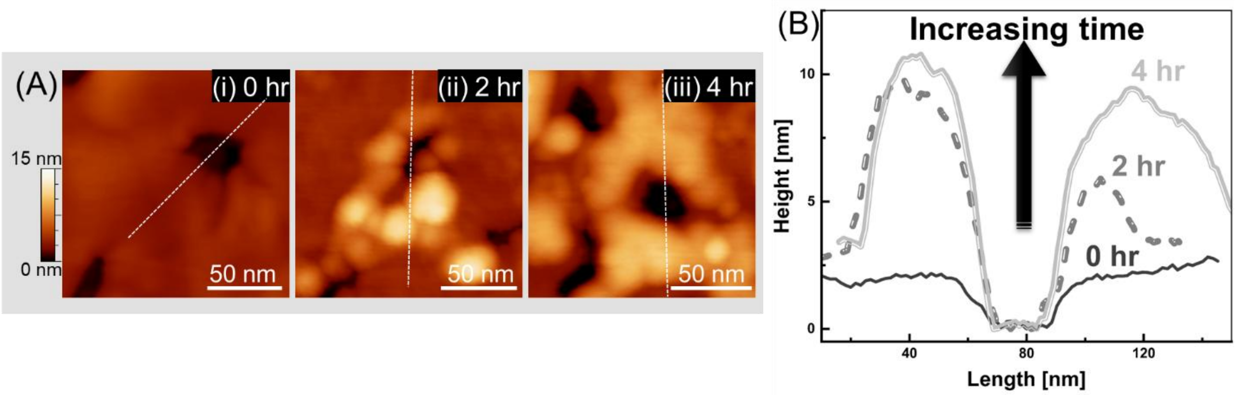

Figure S10. (A) The AFM images of a pore of (i) an unmodified, (ii) 2 hr, and (iii) 4 hr polymerized porous graphene. (B) The evolution of the surface profiles of a pore with polymerization. The white dashed lines indicate the areas where the surface profiles were calculated. All the scale bars are 50 nm, and the height range is 15 nm.

- **Influence of reaction time on large-scale porous graphene morphology:**

Histogram analysis of the AFM images at varying polymerization time, is also consistent with the observed surface profiles (see Figure S11). Until 2 hr polymerization time, we can identify the peak associated with the basal plane at *ca.* 2.5 nm. At 4 hours of polymerization time, we observe a slight shift in the peak at *ca.* 3.2 nm and additionally, we can clearly observe a distinct tail that is resulting from the grafted polystyrene chains. Further polymerization leads to a significant broadening of the peak associated with basal plane, implying that the polymers are randomly oriented covering the underlying surface due to extended polymerization. Hence, we can conclude that the growth kinetics of polystyrene in this graphene sample is also comparable to what we observed for type 1 graphene.

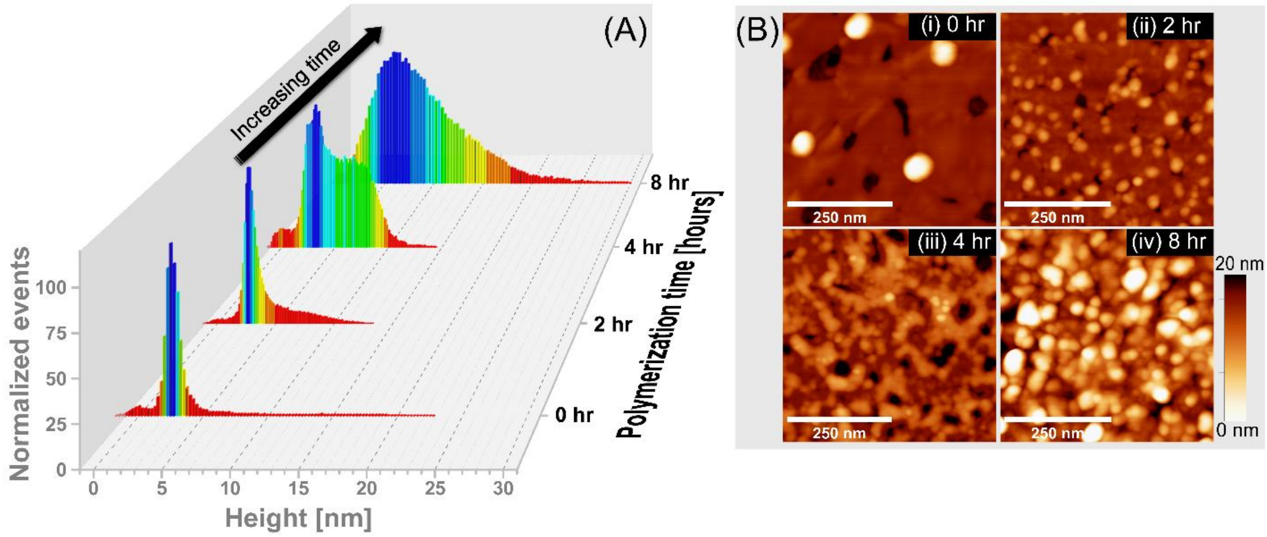

Figure S11. (A) The histogram of the AFM images measured at varying polymerization times. The number of structures with height of > 10 nm increases with increasing polymerization time. (B) Overview AFM images of porous graphene (i) before and after (ii) 2 hr (iii) 4 hr, and (iv) 8 hr of SIPGP with styrene monomer. All the scale bars all of 250 nm and the height range is 20 nm.

- **Influence of reaction time on average thickness and rms roughness of the layer:**

From the AFM images we also calculated h_a and R_{rms} in a similar way and the measured data was quite consistent until 12 hr of polymerization. The height analysis indicated a saturation in the thickness of the grafted polymer after 12 hrs of polymerization.

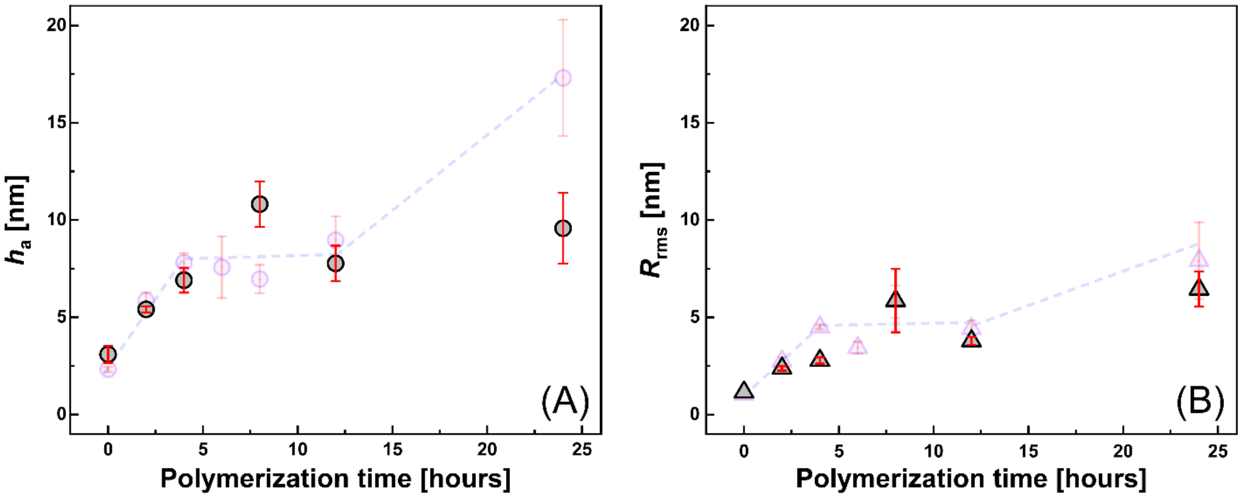

Figure S12. (A) Influence of the polymerization time on average thickness of the graphene layer of two types (type 1 = violet, type 2 = grey). (B) Changes in the root mean square roughness of the graphene layer of two types (type 1 = violet, type 2 = grey) with increasing polymerization time.

S8. The morphology of the layer when carried out on a pristine graphene layer without pores compared to porous graphene:

- **Growth kinetics of SIPGP with styrene on non-porous graphene surfaces:**

To understand the differences in the SIPGP process when it is carried out on a surface with structural defects[3,4] as opposed to porous graphene, we used a pristine graphene surface for the SIPGP process with styrene monomer. SIPGP processing conditions and the transfer of graphene were identical to that used for porous graphene samples. In a pristine graphene with no pores, the "defects" reactive to styrene polymerization are random. The defects can be structural in the basal plane[3,4] or from the grain boundaries.[17] Hence, the polymerization process should also be random resulting in a generalized increase in thickness of the basal plane. The large-scale AFM images (Figure S13) of 4 hr and 12 hr polymerized non-porous graphene suggest the stochastic nature of the grafting process where the grafting areas are not defined. Furthermore, unlike the porous graphene here, the basal plane thickening becomes evident even at 4 hr polymerization time. Consistent with previous works on SIPGP of graphene,[2,18] we can identify a carpet of polymer on the graphene surface resulting in a significant basal plane thickening as evidenced by the histogram analysis shown in Figure S12 (the peak of the histogram shifts from 0.94 nm (0 hr) to 6.4 nm (12 hr)). Furthermore, at any point (especially at low polymerization times), we could identify only one broad peak in the histogram rather than two distinct peaks associated with the basal plane and the grafting region observed for porous graphene type 1 and 2 (Figure 3 and Figure S11). This observation again emphasizes the specificity of the SIPGP process when carried out on graphene surfaces with pre-defined defects.

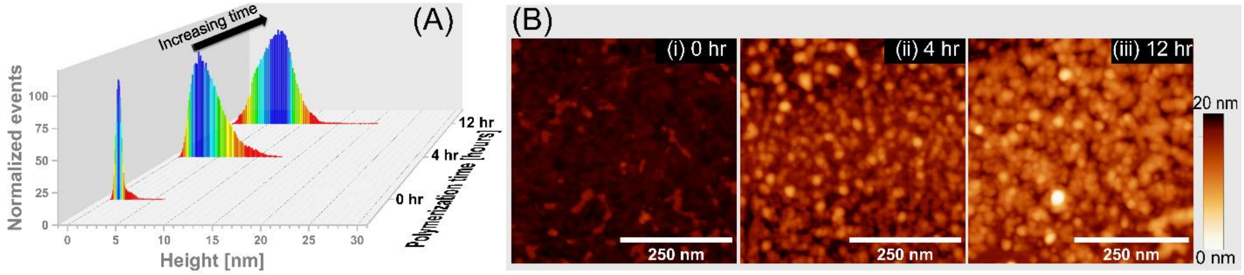

Figure S13. (A) Histogram analysis of AFM images obtained at varying polymerization times of the pristine graphene without any pores. The unmodified graphene shows a peak at 0.93 nm indicating the presence of single layer graphene. The small tail observed in the histogram is attributed to the presence of localized PMMA residue from the transfer process of single layer graphene. With 4 hr of polymerization, we can observe a significant broadening of the peak with a shift towards higher thickness (from 0.93 nm to 2.5 nm), indicating a non-site-specific functionalization of the polystyrene to the graphene film. Further polymerization broadens the peak further with the maxima of the peak being at 6.4 nm. B) Overview AFM images of single layer non-porous graphene (i) before and after (ii) 4 hr, and (iii) 12 hr of SIPGP with styrene monomer. All the scale bars are 250 nm, and the height range is 20 nm.

- **Comparison of grafted regions of polystyrene in both porous and non-porous films:**

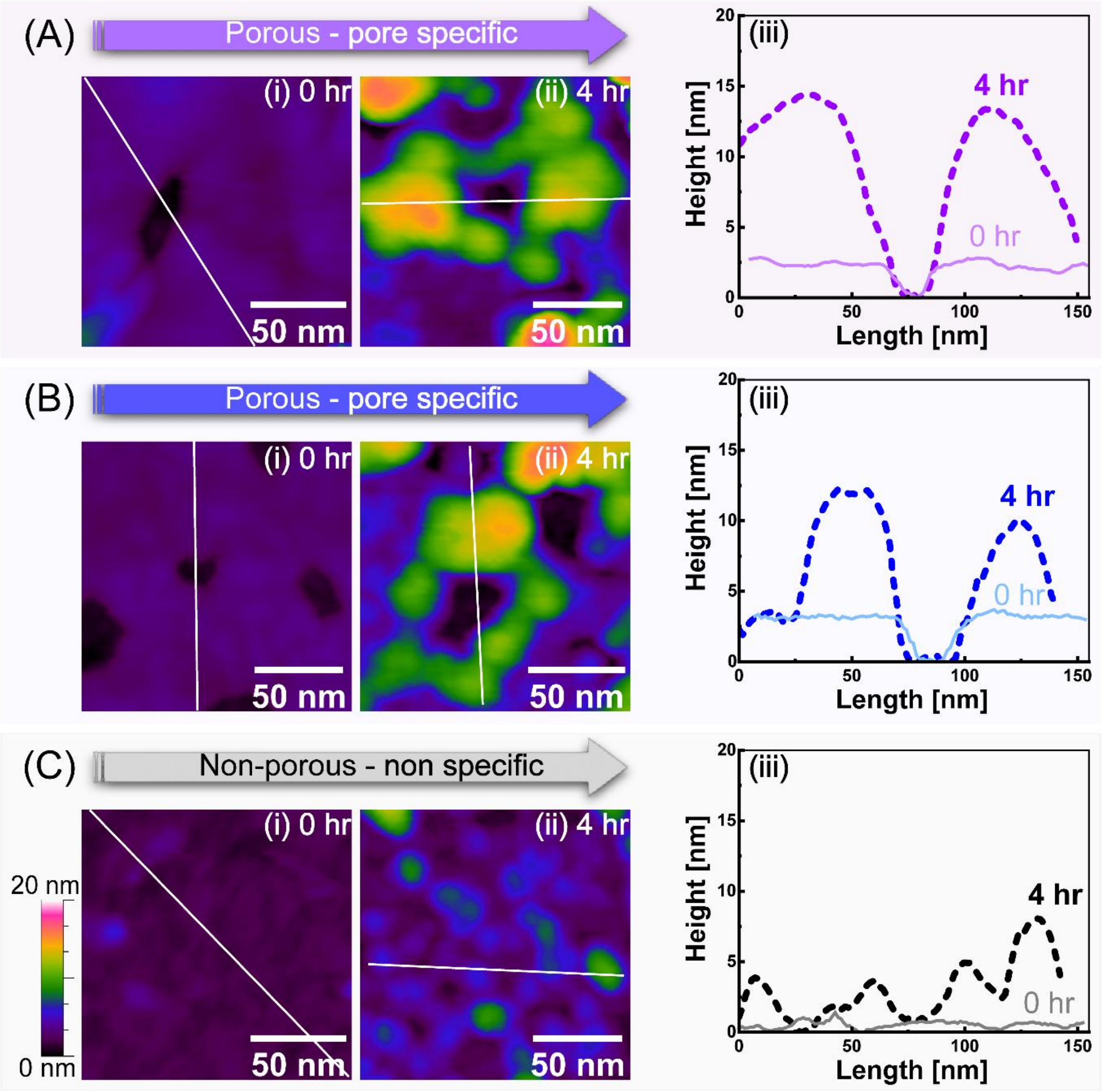

Figure S14. Influence of defined defects on the grafting regions. (A) AFM images of which focuses on the pores of type 1 porous graphene (i) with no SIPGP, (ii) with 4 hr of SIPGP , and (iii) the surface profiles (measured along the white lines) highlight the changes in the pore morphology associated with the polymerization process. (B) AFM images of the pores of type 2 porous graphene (i) with no polymerization, (ii) with 4 hr of SIPGP, and (iii) the surface profiles of the pores (measured along the white lines shown in the images) demonstrate the propensity of the polymers to be grafted along the pores. (C) AFM images of the non-porous graphene (i) with no polymerization, (ii) with 4 hr of SIPGP process, and (iii) the surface profiles of the modified and unmodified graphene (along the white lines drawn in the images) demonstrates the stochastic process of grafting in a surface with undefined defects. All scale bars are 50 nm, and the height range is 20 nm.

S9. Influence of UV light exposure and polymerization on the graphene

To investigate the influence of both polymerization and UV exposure on the overall graphene quality (in terms of defect types/sites) we conducted Raman analysis of the graphene layer (both type 1 and 2) after varying SIPGP times (0 hr, 4 hr, and 24 hr) and after 24 hr of only UV light exposure (type 1 graphene). The measured Raman spectra for both types are represented in Figure S15. The Raman spectra showed no significant change in either I_D/I_G ratios or $I_D/I_{D'}$ ratios. No significant deviations in the intensity ratios indicate that no additional defects are introduced during the process, and further, it alleviates the presence of physiosorbed polymers on the graphene surface. This observation is consistent with Jordan and co-workers' reported work[2].

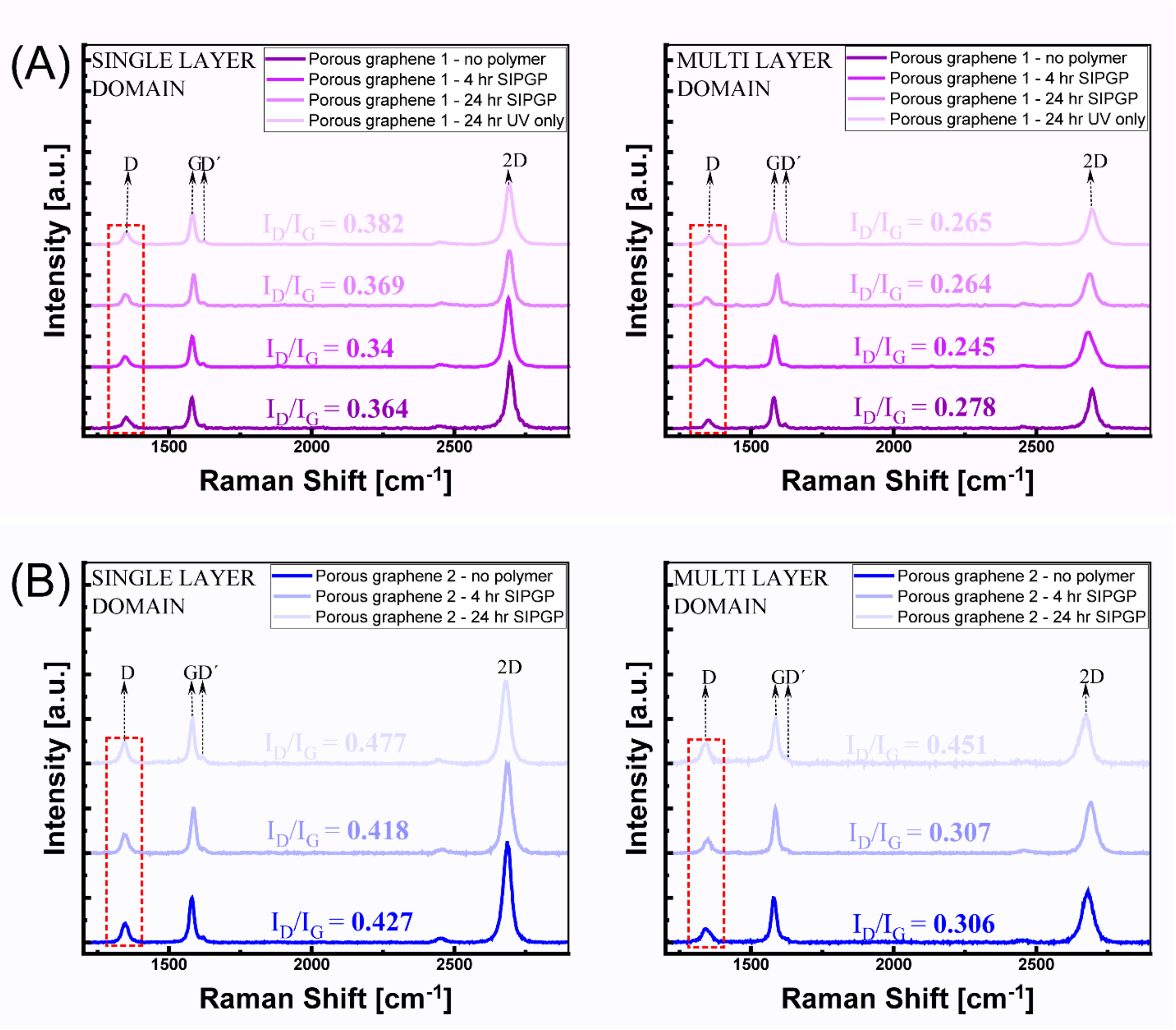

Figure S15. (A) Raman spectra obtained from both single layer and multi-layer graphene domains present in type 1 porous graphene with or without UV exposure and with varying SIPGP times. (B) Raman spectra obtained from both single layer and multi-layer graphene domains of type 2 porous graphene with varying SIPGP times.

S10. Additional histogram analysis of large-scale area:

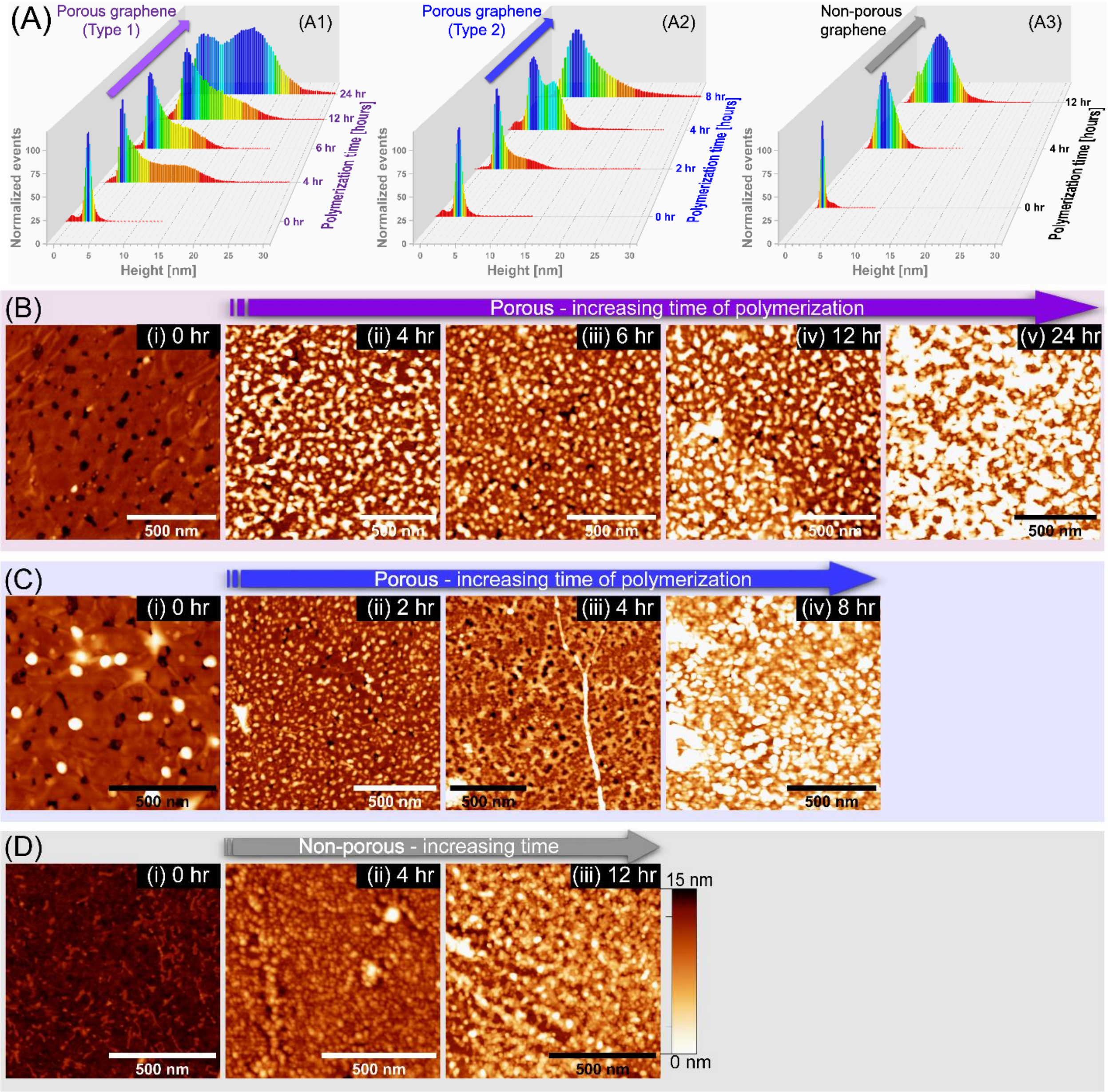

Figure S16. (A) Evolution of histogram with polymerization process for (A1) porous graphene type 1 (violet, from no SIPGP to 24 hr of SIPGP), (A2) porous graphene type 2 (blue, from no SIPGP to 8 hr of SIPGP), and (A3) non-porous graphene (grey, from no SIPGP to 12 hr of SIPGP). (B) Large scale AFM images of porous graphene type 1 of (i) no SIPGP, (ii) 4 hr, (iii) 6 hr, (iv) 12 hr, and (v) 24 hr of SIPGP. (C) Large scale AFM images of porous graphene type 2 of (i) no SIPGP, (ii) 2 hr, (iii) 4 hr, and (iv) 8 hr of SIPGP. (D) Large scale AFM images of non-porous of (i) no SIPGP, (ii) 4 hr, and (iii) 12 hr of SIPGP. All scale bars are 500 nm, and the height range is 15 nm.

S11. Additional pore surface profile analysis:

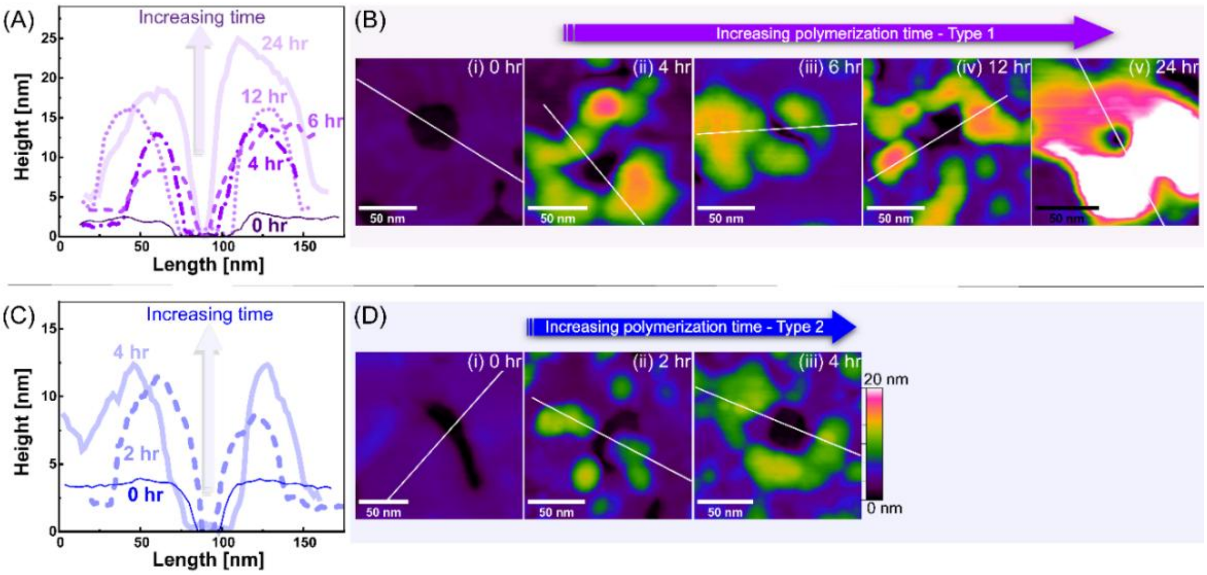

Figure S17. (A) Surface profile analysis of the pores of porous graphene with increasing time of polymerization (0 – 24 hr). (B) AFM images of focusing on the pores of porous graphene type 1 with (i) 0 hr, (ii) 4 hr, (iii) 6 hr, (iv) 12 hr, and (v) 24 hr of SIPGP. (C) Surface profile analysis of the pores of porous graphene with increasing time of polymerization (0 – 4 hr). (B) AFM images of focusing on the pores of porous graphene type 2 with (i) 0 hr, (ii) 2 hr, and (iii) 4 hr of SIPGP. All the scale bars are 50 nm, and the height range is 20 nm.

S12. Comparison of photomasking/carbon deposition *vs* site specific SIPGP:

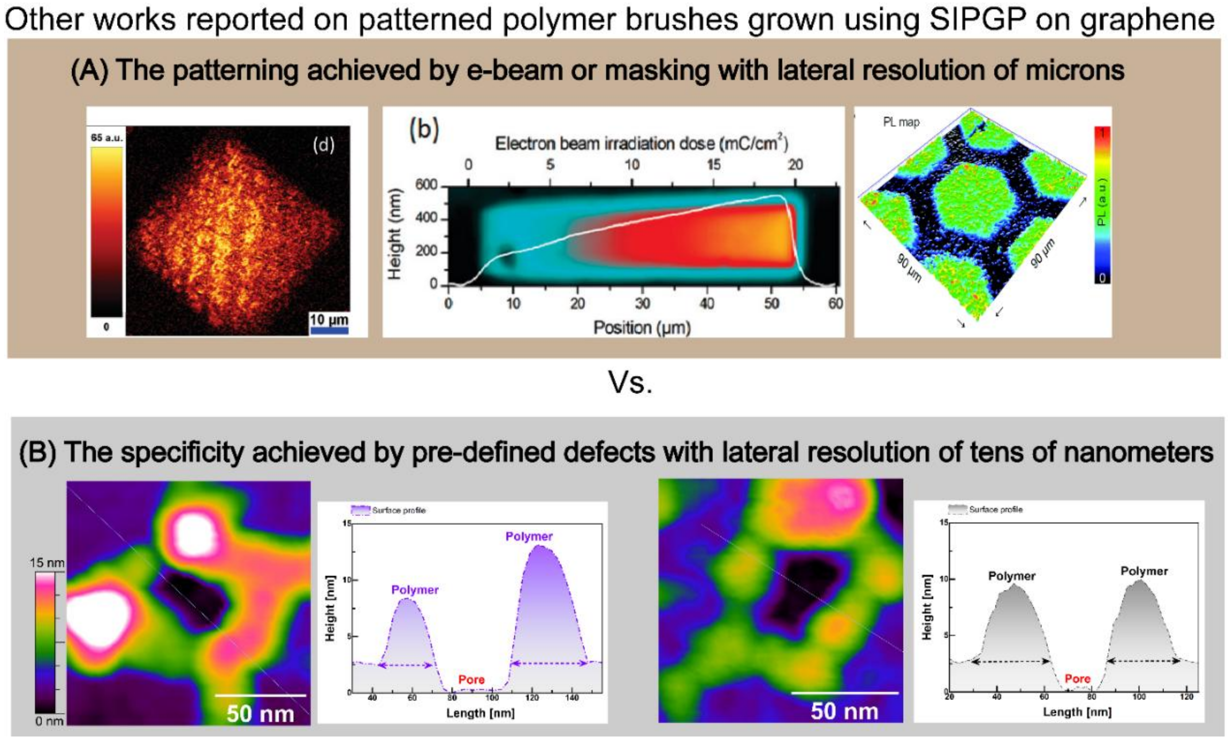

Figure S18. Comparison of the lateral resolution of patterned polymer layers achieved by the SIPGP process to the lateral resolution achieved in this study with the identical process where predefined defects was exploited in a systematic manner. (A) **Top left:** Patterned polymer layers of polystyrene on graphene reported in Steenackers et. al.[2] Reprinted with permission from {Polymer Brushes on Graphene, M. Steenackers, A. M. Gigler, N. Zhang, F. Deubel, M. Seifert, L. H. Hess, C. H. Y. X. Lim, K. P. Loh, J. A. Garrido, R. Jordan, M. Stutzmann, I. D. Sharp, *J. Am. Chem. Soc.* **2011**, 133, 10490}.[2] Copyright {2011} American Chemical Society. **Top-middle:** patterned polymer brushes of variable thicknesses of poly(METAC) brush layer formed on an electron beam-deposited carbon gradient on few layer graphene on SiC reported also in Steenackers *et. al*.[2] Reprinted with permission from

{Polymer Brushes on Graphene, M. Steenackers, A. M. Gigler, N. Zhang, F. Deubel, M. Seifert, L. H. Hess, C. H. Y. X. Lim, K. P. Loh, J. A. Garrido, R. Jordan, M. Stutzmann, I. D. Sharp, *J. Am. Chem. Soc.* **2011**, 133, 10490}.[2] Copyright {2011} American Chemical Society. **Top right:** patterned polystyrene-p3HT carpet reported by Tao Zhang *et. al*.[18] Used with permission of [Royal Society of Chemistry], from [Bottom-up fabrication of graphene-based conductive polymer carpets for optoelectronics, T. Zhang, R. D. Rodriguez, I. Amin, J. Gasiorowski, M. Rahaman, W. Sheng, J. Kalbacova, E. Sheremet, D. R. T. Zahn, R. Jordan, *J. Mater. Chem. C*, *6*, 4919, **2018**]; permission conveyed through Copyright Clearance Center, Inc. In all cases, the lateral resolution of patterning was of the order of microns, due to the resolution-limitation of the pre-processing steps used in these studies. (B) Polymer domains attached along the pore edges reported in our work and the surface profiles along the lines show a lateral resolution of sub-micron scale (50 – 100 nm), differentiating this work clearly from the other fascinating works reported in the field of photo-polymerization. The scale bars in the images (B) are 50 nm and the height range is 15 nm.

S13. Hydrostatic pressure measurements:

Before and after modification, graphene layers were transferred onto Silicon Nitride membranes (SiNx) for the measurements after the etching process. Each SiNx holey frames contain 64 holes (8 x 8) with approximately six µm diameter, where the graphene layers remain free-standing. After the transfer process, each film was characterized with optical and scanning electron microscopy to determine the defect-free graphene-covered regions on the chips. Before the measurement, the graphene-free regions and the transfer-induced defect areas (large cracks on the unmodified graphene) were sealed with epoxy glue. The SiNx membrane was placed onto a sealed chamber, and the top side was carefully filled with DI water without any bubbles forming. The sealing was achieved by placing O-rings on either side of the membrane. After filling the top side completely with water, the chamber was sealed, and pressure (100 mbar to 3 bar) was applied incrementally to the water-filled side *via* a Mass Flow Controller (MFC). The emergence of water leakage with increasing pressure was then visually observed from the backside of the chamber, and the pressure was noted.

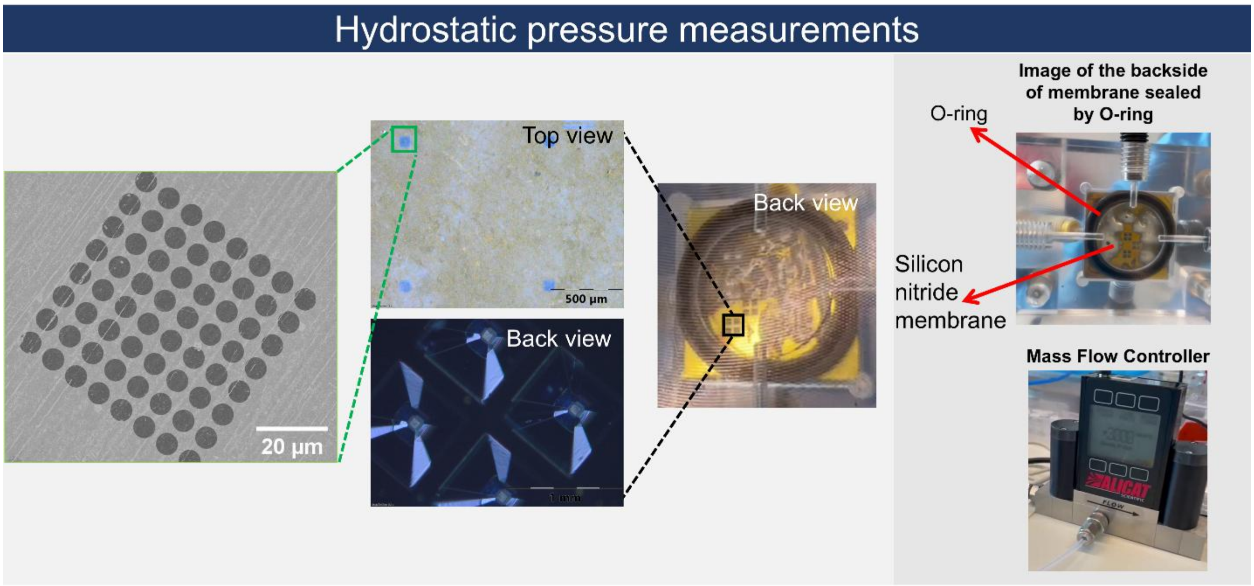

Figure S19. Figure showing the SEM image of the graphene layer transferred onto a SiNx membrane for hydrostatic measurement. Additionally, different components of the measurement process are shown in this figure. Specifically, the chamber used for measuring the water column resistance and the MFC used for applying pressure.

References:

[1] K. Choi, A. Droudian, R. M. Wyss, K.-P. Schlichting, H. G. Park, *Sci. Adv.* **2018**, *4*, eaau0476.

[2] M. Steenackers, A. M. Gigler, N. Zhang, F. Deubel, M. Seifert, L. H. Hess, C. H. Y. X. Lim, K. P. Loh, J. A. Garrido, R. Jordan, M. Stutzmann, I. D. Sharp, *J. Am. Chem. Soc.* **2011**, *133*, 10490.

[3] F. Banhart, J. Kotakoski, A. V Krasheninnikov, *ACS Nano* **2011**, *5*, 26.

[4] Z. Komeily-Nia, L.-T. Qu, J.-L. Li, *Small Sci.* **2021**, *1*, 2000026.

[5] J. Shen, D. Zhang, F.-H. Zhang, Y. Gan, *Appl. Surf. Sci.* **2017**, *422*, 482.

[6] I. Horcas, R. Fernández, J. M. Gómez-Rodríguez, J. Colchero, J. Gómez-Herrero, A. M. Baro, *Rev. Sci. Instrum.* **2007**, *78*, 13705.

[7] M. Seifert, A. H. R. Koch, F. Deubel, T. Simmet, L. H. Hess, M. Stutzmann, R. Jordan, J. A. Garrido, I. D. Sharp, *Chem. Mater.* **2013**, *25*, 466.

[8] J. Díaz, G. Paolicelli, S. Ferrer, F. Comin, *Phys. Rev. B* **1996**, *54*, 8064.

[9] H. Jung, K. T. Park, M. N. Gueye, S. H. So, C. R. Park, *Int. J. Hydrogen Energy* **2016**, *41*, 5019.

[10] A. Bellunato, H. Arjmandi Tash, Y. Cesa, G. F. Schneider, *ChemPhysChem* **2016**, *17*, 785.

[11] X. Wang, S. M. Tabakman, H. Dai, *J. Am. Chem. Soc.* **2008**, *130*, 8152.

[12] Z. Wang, B. Yang, Y. Wang, Y. Zhao, X. M. Cao, P. Hu, *Phys. Chem. Chem. Phys.* **2013**, *15*, 9498.

[13] R. Sharma, J. H. Baik, C. J. Perera, M. S. Strano, *Nano Lett.* **2010**, *10*, 398.

[14] D. W. Boukhvalov, M. I. Katsnelson, *Nano Lett.* **2008**, *8*, 4373.

[15] L. M. Malard, M. A. Pimenta, G. Dresselhaus, M. S. Dresselhaus, *Phys. Rep.* **2009**, *473*, 51.

[16] A. Eckmann, A. Felten, A. Mishchenko, L. Britnell, R. Krupke, K. S. Novoselov, C. Casiraghi, *Nano Lett.* **2012**, *12*, 3925.

[17] X. Fan, S. Wagner, P. Schädlich, F. Speck, S. Kataria, T. Haraldsson, T. Seyller, M. C. Lemme, F. Niklaus, *Sci. Adv.* **2024**, *4*, eaar5170.

[18] T. Zhang, R. D. Rodriguez, I. Amin, J. Gasiorowski, M. Rahaman, W. Sheng, J. Kalbacova, E. Sheremet, D. R. T. Zahn, R. Jordan, *J. Mater. Chem. C* **2018**, *6*, 4919.
